# Changes in the Quality Characteristics of Gastrodia Elata During Traditional Processing: Insights from Multi-Omics Integration

**DOI:** 10.3390/foods15152704

**Published:** 2026-07-31

**Authors:** Weiqian Wang, Jia Liu, Muhammad Aaqil, Siyu Zhou, Xinyu Wei, Linyu Li, Zengzeng Chen, Jun Sheng, Yang Tian, Cunchao Zhao

**Affiliations:** 1 College of Food Science and Technology, Yunnan Agricultural University, Kunming 650201, China; 19982468477@163.com (W.W.); aaqilkhan26@gmail.com (M.A.); zzz121300y@163.com (S.Z.); 15696400575@163.com (X.W.); 18387988813@163.com (L.L.); 15096767609@163.com (Z.C.); shengjunpuer@163.com (J.S.); 2 Yunnan International Joint Laboratory of China-Cambodia Signiture Agro-Products Green Development, Kunming 650201, China; 3 College of Science, Yunnan Agricultural University, Kunming 650201, China; jane-3223505@163.com; 4 Yunnan Key Laboratory of Precision Nutrition and Personalized Food Manufacturing, Yunnan Agricultural University, Kunming 650201, China; 5 Yunnan Plateau Characteristic Agricultural Industry Research Institute, Kunming 650201, China

**Keywords:** *Gastrodia elata*, traditional processing, water distribution, quality characteristics, multi-omics

## Abstract

*Gastrodia elata Bl*. (GE), a representative medicine–food homology material, requires appropriate processing prior to food application to meet established medicinal quality standards. However, the effects of different traditional processing methods on its quality attributes remain insufficiently elucidated. In this study, three representative processing methods, steaming, moistening, and bran-frying, were systematically compared to evaluate their impact on the structural, compositional, and flavor characteristics of GE. The results demonstrated that processing markedly altered both the physical structure and chemical composition of GE. Moistening caused minimal disruption to the native tissue architecture and largely preserved the free-water distribution pattern. Notably, gastrodin and p-hydroxybenzyl alcohol contents reached 0.38% and 4.16%, respectively, the highest among all treatments, while the overall flavor profile remained closest to that of fresh GE. In contrast, bran-frying induced pronounced dehydration and structural densification, with volatile compounds and differential metabolites accounting for 72.83% and 41.01%, respectively. This treatment also exhibited elevated levels of total phenolics, flavonoids, and protein. Steaming, by comparison, promoted starch gelatinization and water redistribution, shifting the flavor profile from a delicate herbal note toward enhanced umami, floral, and mildly roasted characteristics. Overall, these findings reveal that processing-induced structural remodeling and changes in water state play critical roles in regulating the quality attributes of GE. By integrating structural characterization, water-state analysis, and multi-omics data, this study provides a systematic understanding of the formation of GE quality attributes under different processing methods, offering a theoretical basis for quality control and process optimization.

## 1. Introduction

*Gastrodia elata Bl*. (GE) is primarily distributed in southwestern China, particularly in Yunnan and Guizhou, and is widely recognized as a representative traditional East Asian medicinal herb. In traditional medicine, GE has long been used to alleviate dizziness, epilepsy, and other neurological disorders [1]. Beyond its medicinal applications, GE also plays an important role in dietary culture and is commonly incorporated into tonic soups and medicinal cuisines. Notably, in November 2023, GE was officially included in the Medicine Food Homology (MFH) catalogue issued by the National Health Commission of the People’s Republic of China, marking a significant transition from clinical use toward broader applications in the health-food sector. Modern pharmacological studies have further demonstrated that GE is rich in gastrodin (GAS), p-hydroxybenzyl alcohol (HBA), and flavonoids and exhibits diverse bioactivities, including neuroprotective effects (e.g., amelioration of Parkinson’s disease and cognitive impairment), modulation of gut microbiota, and attenuation of oxidative stress [2,3].

Prior to clinical application or dietary development, MFH materials typically undergo a series of processing procedures collectively referred to as “Paozhi” in traditional Chinese medicine, including cleansing, cutting, and specific heat- or adjuvant-assisted treatments [4]. From the perspectives of modern food science and pharmacology, “Paozhi” represents not only a critical step for standardizing the quality of medicinal materials, but also a complex process involving structural transformation and chemical conversion. Through precise regulation of processing parameters such as temperature, humidity, and pressure, traditional “Paozhi” techniques can profoundly remodel the microstructure of medicinal materials. This remodeling plays a key role in altering their physicochemical properties and bioactivity. These effects are further influenced by interactions with auxiliary materials, such as wheat bran and rice wine. These changes include alterations in cell wall permeability, starch crystalline transitions, and protein denaturation, which in turn enhance the release efficiency of bioactive compounds or promote the degradation of potentially harmful components, thereby achieving an optimal balance among safety, efficacy, and sensory quality.

In recent years, increasing attention has been directed toward the influence of processing technologies on the quality attributes of MFH materials. For instance, ref. [5] investigated the effects of post-processing drying on GE from the perspective of drying kinetics; ref. [6] demonstrated that bran-frying modifies the polysaccharide structure of Atractylodes lancea, thereby enhancing its efficacy in alleviating intestinal injury. Similarly, ref. [4] employed the traditional “nine-steaming, nine-drying” process for *Polygonatum kingianum* and dynamically monitored compositional changes, elucidating the mechanisms by which processing reduces tingling sensation while enhancing antioxidant activity.

Due to its high moisture and polysaccharide contents, fresh GE is highly susceptible to enzymatic browning and exhibits poor storage stability [7]. Consequently, traditional processing is essential for enzyme inactivation and tissue stabilization prior to further utilization. According to the Chinese Pharmacopoeia (2020 edition) and traditional practices, steaming, moistening, bran-frying, and ginger-processing are the primary methods applied to GE. Among these, steaming effectively inactivates enzymes and improves tissue texture; however, the associated high-temperature and high-humidity conditions may increase moisture content and induce extensive starch gelatinization, leading to a sticky texture, increased susceptibility to spoilage, and the need for subsequent drying [5]. Moistening, in contrast, is a milder process in which controlled water absorption softens the material, facilitating subsequent slicing while preserving active constituents and minimizing efficacy loss [8]. Bran-frying, as a typical dry-heat treatment, imparts distinctive aroma and color through heat transfer from wheat bran and Maillard reactions; ref. [9] reported that this process significantly reduces volatile oil content while reshaping the chemical composition of Atractylodes lancea, thereby enhancing its hepatoprotective effects.

Despite these advances, existing studies on GE have primarily focused on drying optimization and individual component analysis, whereas systematic investigations into the effects of different “Paozhi” methods on flavor evolution and their underlying chemical mechanisms remain limited.

Therefore, in this study, three representative processing methods, steaming (GE-stm), moistening (GE-ms), and bran-frying (GE-wbf), were selected to comprehensively evaluate their effects on the quality attributes of GE. Fresh GE and the three processed samples were comparatively evaluated to determine how processing-induced changes in tissue microstructure and water distribution were associated with variations in major chemical constituents and instrument-derived odor and taste response patterns. Scanning electron microscopy and low-field nuclear magnetic resonance were used to characterize microstructural morphology and water migration, while high-performance liquid chromatography and spectrophotometric assays were employed to quantify gastrodin, p-hydroxybenzyl alcohol, starch, soluble polysaccharides, total phenolics, total flavonoids, and protein. Electronic nose and electronic tongue analyses were used to characterize the overall odor and taste responses, whereas HS-SPME-GC × GC-TOF-MS and UPLC-MS/MS were applied to profile volatile compounds and non-volatile metabolites, respectively. By integrating these datasets, this study sought to identify processing-specific quality patterns and clarify the relationships among structural remodeling, water-state transformation, chemical composition, and flavor formation. The findings provide a scientific basis for quality control, process optimization, and the selection of appropriate processing methods for the high-value food utilization of GE as a Medicine Food Homology material.

## 2. Materials and Methods

### 2.1. Materials

Fresh *Gastrodia elata* (GE) samples used in this study were harvested from Zhaotong City, Yunnan Province, China. The samples were thoroughly washed to remove surface impurities and subsequently stored at 4 °C until further use. Prior to experimentation, the samples were equilibrated to room temperature. The initial average moisture content was determined to be 80.72% (wet basis, w.b.) using a fully automated rapid halogen moisture analyzer (AKD-W10S, Wuxi, China). Reference standards for high-performance liquid chromatography (HPLC) analysis included gastrodin (GAS, purity ≥ 98%) and p-hydroxybenzyl alcohol (HBA, purity ≥ 98%). Chromatography-grade methanol was obtained from Sigma-Aldrich (St. Louis, MO, USA). Standards and key reagents used for the determination of starch, polysaccharides, total phenolics, total flavonoids, and protein included glucose, gallic acid, bovine serum albumin (BSA), phenol, concentrated sulfuric acid, and Folin–Ciocalteu reagent. All other chemicals, including anhydrous ethanol and anhydrous sodium carbonate, were of analytical grade (AR) and purchased from Aladdin Bio-Chem Technology Co., Ltd. (Shanghai, China). Ultrapure water (resistivity ≥ 18.2 MΩ · cm) was used throughout all experiments.

### 2.2. Preparation of GE

Fresh GE samples from the same batch, exhibiting uniform maturity and similar size, were selected for this study. The steam-softening and slicing method was first applied according to the procedure reported by [5]. Briefly, GE samples were steamed with saturated steam at 100 °C under atmospheric pressure for 20 min. Following steaming, the samples were sliced into approximately 5 mm thick pieces and evenly spread in a hot-air drying oven (DHG-9140A, Yiheng Scientific Instrument Co., Ltd., Shanghai, China). The drying temperature was set at 50 °C, and the samples were weighed at 30 min intervals until the moisture content decreased to approximately 15%. The samples obtained were designated as GE-stm. For the moistening-softening and slicing treatment, 40 mL of water was evenly sprayed onto 100 g of GE, followed by a moistening process for 24 h. During this period, the samples were turned every 30 min to ensure uniform water absorption [10]. After moistening, surface moisture was gently removed, and the samples were sliced into pieces approximately 5 mm thick. The slices were then dried under the same conditions as described above to obtain GE-ms. Bran-frying was conducted according to the method described by [6]. Briefly, fresh GE samples were first sliced into approximately 5 mm thick pieces. Subsequently, 150 g of wheat bran was heated in a wok until smoking, after which 1000 g of GE slices was immediately added and stir-fried rapidly over medium heat. When the surface of the GE slices turned dark yellow, the samples were removed, sieved to eliminate residual bran, and allowed to cool to room temperature, yielding the GE-wbf samples.

### 2.3. Microscopic Structure Analysis of GE

#### 2.3.1. Low-Field Nuclear Magnetic Resonance (LF-NMR)

The LF-NMR analysis was performed according to the method described by [11]. The nuclear magnetic resonance signals of GE slices were measured using an LF-NMR spectrometer (MesoMR23-060H–I, Niumag, Shanghai, China). Measurements were conducted at a proton resonance frequency of 20 MHz and a temperature of 35 °C using the Carr–Purcell–Meiboom–Gill (CPMG) pulse sequence. The resulting transverse relaxation time (T_2_) data were subsequently analyzed to characterize the water distribution within the samples. LF-NMR was used to characterize proton transverse relaxation behavior and water distribution rather than to identify or quantify individual chemical compounds. Therefore, commercial reference standards were not applicable to this analysis.

#### 2.3.2. Scanning Electron Microscope (SEM)

Scanning electron microscopy (SEM) analysis was conducted according to the method reported by [12]. The surface microstructure of GE samples was observed using a ZEISS Sigma 360 scanning electron microscope (Carl Zeiss, Oberkochen, Germany). Microstructural images were acquired at magnifications of 100× and 500×.

### 2.4. Determination of Chemical and Bioactive Components in GE

#### 2.4.1. Determination of Gastrodin and p-Hydroxybenzyl Alcohol

The contents of gastrodin (GAS) and p-hydroxybenzyl alcohol (HBA) in GE were determined by high-performance liquid chromatography (HPLC) with slight modifications to the method described by [13]. Briefly, approximately 0.5 g of sample powder, passed through a 65-mesh sieve, was accurately weighed and extracted with 25 mL of 50% ethanol by ultrasonication at 500 W and 40 kHz for 30 min. After cooling to room temperature, the extract was reweighed to compensate for solvent loss, thoroughly mixed, and filtered, followed by centrifugation at 13,800× *g* for 10 min. The supernatant was subsequently filtered through a 0.22 μm membrane to obtain the final sample solution.

Chromatographic analysis was performed using a ZORBAX SB-C18 column (4.6 mm × 250 mm, 5 μm; Agilent Technologies, Palo Alto, CA, USA). The mobile phase consisted of 0.05% phosphoric acid aqueous solution (A) and acetonitrile (B), applied under gradient elution conditions. The flow rate was set at 0.8 mL/min, the column temperature was maintained at 30 °C, the injection volume was 10 μL, and detection was performed at 220 nm. Mixed standard solutions of GAS and HBA were prepared using the corresponding commercial reference standards, and quantification was performed using the external standard method.

#### 2.4.2. Determination of Soluble Polysaccharide Content

The soluble polysaccharide content of GE was determined using the phenol–sulfuric acid method according to the procedure described by [14], with glucose used as the reference standard.

#### 2.4.3. Determination of Total Phenolic Content

The total phenolic content of GE was determined using the Folin–Ciocalteu colorimetric method. Briefly, 0.10 g of dried sample powder was accurately weighed into a 2 mL centrifuge tube, mixed with 1 mL of ethanol, and extracted with continuous shaking at 60 °C for 2 h. The extract was centrifuged at 12,000 rpm for 10 min, and the resulting supernatant was collected for analysis.

For the colorimetric reaction, 10 μL of the supernatant was added to both the test and sample-control wells. Subsequently, 50 μL of Folin–Ciocalteu reagent was added to the test well, whereas 50 μL of distilled water was added to the sample-control well. After mixing and incubation at room temperature for 2 min, 50 μL of Na_2_CO_3_ solution and 90 μL of distilled water were added. The reaction mixture was incubated at room temperature for 10 min, and absorbance was measured at 765 nm using a Multiskan GO microplate reader (Thermo Fisher Scientific, Waltham, MA, USA). Ethanol was used as the reagent blank, and gallic acid was used to construct the calibration curve. The results were expressed as milligrams of gallic acid equivalents per gram of sample dry weight (mg GAE/g DW).

#### 2.4.4. Determination of Total Flavonoid Content

The total flavonoid content of GE was determined using a commercial plant flavonoid colorimetric assay kit based on the formation of a colored complex between flavonoids and aluminum ions. The samples were first dried to a constant weight, ground into powder, and passed through a 40-mesh sieve. Approximately 0.05 g of the dried powder was accurately weighed and extracted with 1 mL of 60% ethanol by continuous shaking at 60 °C for 2 h. The extract was centrifuged at 10,000× *g* and 25 °C for 10 min, and the supernatant was collected for subsequent analysis.

Briefly, 80 μL of the sample supernatant was mixed with 20 μL of reagent 1 and incubated at 25 °C for 6 min. Subsequently, 20 μL of reagent 2 was added, followed by incubation at 25 °C for another 6 min. Finally, 80 μL of reagent 3 was added, and the mixture was incubated at 25 °C for 15 min. Absorbance was measured at 510 nm using a Multiskan GO microplate reader (Thermo Fisher Scientific, USA). For the blank, 80 μL of 60% ethanol was used instead of the sample extract and subjected to the same reaction procedure. Total flavonoid content was calculated from the corresponding calibration curve and expressed as milligrams per gram of sample dry weight (mg/g DW).

#### 2.4.5. Determination of Protein Content

The protein content of GE was quantified using the bicinchoninic acid assay, with bovine serum albumin used as the reference standard.

#### 2.4.6. Determination of Starch Content

The starch content of GE was determined by enzymatic hydrolysis using thermostable α-amylase and amyloglucosidase. The released glucose was subsequently quantified using glucose oxidase/peroxidase reagent, and absorbance was measured at 510 nm.

### 2.5. Electronic Nose Analysis

Volatile compound analysis was performed using a fast gas chromatography electronic nose system (FOX 3000, Alpha MOS, Toulouse, France), according to the method described by [15]. Briefly, 5.0 g of each sample was accurately weighed into a 50 mL centrifuge tube, with three replicates prepared for each group. The tubes were sealed with parafilm and equilibrated at room temperature for 30 min to allow headspace stabilization.

Volatile compounds were analyzed using a direct headspace sampling method, in which the sampling needle was inserted through the sealing film into the headspace region. The operating parameters were set as follows: sampling frequency, 1 s per cycle; sensor self-cleaning time, 60 s; sample injection time, 5 s; injection flow rate, 400 mL/min; and total acquisition time, 80 s. For data processing, sensor response values recorded between 69 and 71 s were extracted as steady-state signals for subsequent analysis.

### 2.6. Electronic Tongue Analysis

Electronic tongue analysis was conducted with slight modifications based on the method reported by [16], using a taste sensing system (electronic tongue, Insent, Kanagawa, Japan). Briefly, samples were mixed with deionized water at a ratio of 1:20 (*w*/*v*), followed by grinding, homogenization, centrifugation, and filtration. The resulting supernatants were collected for subsequent analysis. Prior to measurement, the sensors were sequentially cleaned in washing solution for 90 s, followed by rinsing in two cups of reference solution for 120 s each, and then allowed to equilibrate for 30 s. During analysis, the sensors were first immersed in the sample solution for 30 s to record the initial taste values, then rinsed for 3 s and transferred into fresh reference solution for aftertaste measurement for an additional 30 s. For the five basic taste sensors and the bitterness sensors, each sample was measured four times. The data from the first cycle were discarded, and the average of the remaining three measurements was used as the result.

### 2.7. HS-SPME-GC×GC-TOF-MS Method for Volatile Compound Profiling

Samples were removed from a −80 °C freezer, ground under liquid nitrogen, and vortex-mixed to ensure homogeneity. Approximately 500 mg of each sample was accurately weighed into a headspace vial, followed by the addition of 2 mL of saturated NaCl solution and 20 μL of internal standard solution (10 μg/mL). After equilibration at 60 °C with shaking for 5 min, a solid-phase microextraction (SPME) fiber (120 μm DVB/CWR/PDMS) was inserted into the vial headspace for adsorption extraction for 15 min. Following extraction, the fiber was introduced into the GC injector and thermally desorbed at 250 °C for 5 min prior to GC × GC-TOF-MS analysis. After each injection, the SPME fiber was conditioned at 270 °C for 10 min to prevent interference from residual compounds in subsequent analyses.

Chromatographic analysis was performed using a LECO Pegasus 4D system (LECO, St. Joseph, MI, USA) coupled with an Agilent 8890A gas chromatography system (Agilent Technologies, Palo Alto, CA, USA), equipped with a split/splitless injector, a dual-stage cryogenic modulator, and a time-of-flight mass spectrometer (TOFMS; LECO Corporation, St. Joseph, MI, USA). Separation was achieved on a DB-Heavy Wax column (30 m × 250 μm × 0.5 μm; Agilent Technologies, Santa Clara, CA, USA), using high-purity helium (>99.999%) as the carrier gas at a constant flow rate of 1.0 mL/min. The oven temperature program was as follows: initial temperature of 50 °C held for 2 min, increased to 240 °C at a rate of 5 °C/min, and maintained at 240 °C for 5 min. Mass spectrometric detection was performed in electron impact (EI) mode at 70 eV. The transfer line and ion source temperatures were both set at 250 °C. Data were acquired at a rate of 10 spectra/s over an *m*/*z* range of 35–550, with the detector voltage set at 1960 V. The GC × GC-TOF-MS analysis was used for comparative volatile profiling rather than absolute quantification. Commercial reference standards were not used for the individual volatile compounds. The numerical values used for statistical analysis represent normalized chromatographic peak areas and indicate the relative abundance of the detected compounds among the sample groups. Therefore, these data are dimensionless and do not represent absolute concentrations. To evaluate the contribution of individual volatile compounds to the overall aroma profile, the relative odor activity value (ROAV) was calculated. Compounds with ROAV ≥ 1 were defined as key aroma compounds contributing decisively to the overall aroma profile, whereas compounds with 0.1 ≤ ROAV < 1 were considered aroma-modifying components [17]. The formula used for ROAV calculation is given below:

(1) $${ROAV}_{i}=100\times \frac{{OAV}_{i}}{{OAV}_{max}}$$

*OAV_max_* represents the highest odor activity value among volatile compounds, and *OAV_i_* is the odor activity value of a particular volatile compound.

(2) $$OAV=\frac{C_{i}}{T_{i}}$$

*C_i_* represents the concentration percentage of a volatile compound in the sample, while *T_i_* represents the odor threshold of the compound in water.

### 2.8. UPLC-MS/MS Method for Non-Volatile Metabolite Profiling/MS

Approximately 50 mg of each sample was accurately weighed and subjected to grinding, sonication, and centrifugation, followed by concentration and drying prior to LC-MS analysis. Liquid chromatography was performed using a Vanquish UHPLC system (Thermo Fisher Scientific, USA) equipped with an ACQUITY UPLC HSS T3 column (2.1 × 100 mm, 1.8 μm; Waters Corporation, Milford, MA, USA). The column temperature was maintained at 40 °C, the flow rate was set at 0.3 mL/min, and the injection volume was 2 μL. For LC-ESI(+)-MS analysis, mobile phase A consisted of 0.1% formic acid in water, and mobile phase B consisted of 0.1% formic acid in acetonitrile. The gradient elution program was as follows: 0–1 min, 10% B; 1–5 min, 10–98% B; 5–6.5 min, 98% B; 6.5–6.6 min, 98–10% B; and 6.6–8 min, 10% B. For LC-ESI(−)-MS analysis, mobile phase A consisted of 5 mM ammonium formate in water, and mobile phase B was acetonitrile. The gradient program was identical to that described above. Mass spectrometric detection was performed using an Orbitrap Exploris 120 mass spectrometer (Thermo Fisher Scientific, USA) equipped with an electrospray ionization (ESI) source and operated in Full MS-ddMS^2^ mode (data-dependent MS/MS). The operating parameters were as follows: sheath gas pressure, 40 arb; auxiliary gas flow, 10 arb; spray voltage, 3.50 kV in positive mode and −2.50 kV in negative mode; capillary temperature, 325 °C; MS^1^ scan range, *m*/*z* 100–1000; MS^1^ resolving power, 60,000 FWHM; MS/MS resolving power, 15,000 FWHM; collision energy, 30%; and dynamic exclusion time, automatic. The UPLC-MS/MS analysis was conducted as an untargeted metabolomic profiling approach rather than an absolute quantitative analysis. Commercial reference standards were not used for the individual detected metabolites. The values used for subsequent statistical analysis represent normalized chromatographic peak areas and indicate relative metabolite abundance. Therefore, these values are dimensionless and should not be interpreted as absolute concentrations.

### 2.9. Statistical Analysis

Statistical analysis was performed using one-way analysis of variance (ANOVA), followed by Tukey’s multiple comparison test, implemented in SPSS version 27.0 (IBM, Armonk, NY, USA). All data are presented as mean ± standard deviation (SD). Graphical representations were generated using OriginPro 2024b (OriginLab, Northampton, MA, USA).

## 3. Results and Discussion

### 3.1. Microscopic Structural Analysis of GE

#### 3.1.1. SEM Analysis

**Figure 1:**
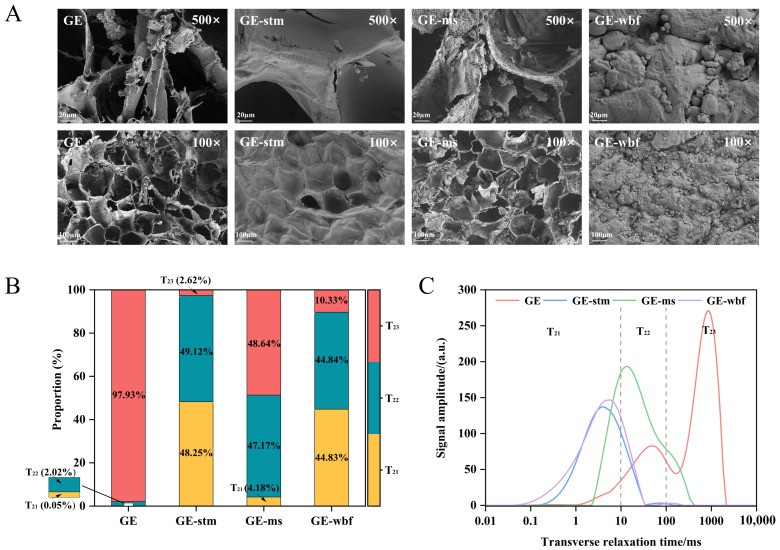
(**A**) Water content map. (**B**) Transverse relaxation time (T2) curves for GE. (**C**) Scanning electron microscope (SEM). GE: Fresh *Gastrodia elata* (GE), GE-stm: Steamed GE, GE-ms: moistened GE, GE-wbf: bran-fried GE.

The microstructure of GE tissue is a key factor governing the distribution and content of its chemical constituents [12]. As shown in Figure 1A, untreated GE exhibited a compact and well-organized cellular structure. At 100× magnification, clear and distinct textures were observed, with well-preserved rounded vascular bundles. At 500× magnification, the cell lumens appeared relatively open, the cell wall surfaces displayed well-defined textures, and numerous starch granules were uniformly distributed on both the inner and outer surfaces of the vascular tissues [5]. Steaming markedly altered the native cellular morphology. At 100× magnification, GE-stm exhibited a blurred honeycomb-like structure with partial fusion of cell boundaries, which may be attributed to the degradation of middle lamella pectin, leading to cell wall rupture and pronounced tissue softening [18]. At 500× magnification, extensive starch gelatinization was observed. Under high-temperature saturated steam, the initially loose intracellular components appeared to melt and reorganize into continuous film-like or massive structures covering the cell surface [19]. In contrast, moistening caused minimal disruption to the microstructure of GE. At 100× magnification, GE-ms largely retained intact cell contours and clearly discernible vascular bundle structures. Although slight shrinkage of the cell walls and uneven distribution of surface starch granules were observed at 500× magnification, the overall structure remained relatively intact, preserving a well-defined porous architecture. The surface morphology of GE after bran-frying exhibited markedly different characteristics. At 100× magnification, the original honeycomb-like porous structure disappeared and was replaced by a dense and irregularly wrinkled layer. At 500× magnification, the surface was covered with numerous compact granular protrusions. These changes are primarily attributed to intense Maillard reactions induced by heat transfer from wheat bran during stir-frying, accompanied by rapid thermal dehydration and substantial consumption of phenolic compounds and polysaccharides [20]. Consequently, a dense crust composed mainly of denatured proteins and gelatinized starch was formed on the tissue surface.

#### 3.1.2. LF-NMR Analysis

LF-NMR is a non-destructive analytical technique widely used to evaluate water distribution, state transitions, and mobility during food processing [21]. The transverse relaxation time (T_2_) of hydrogen protons in GE reflects the binding strength between water and the matrix and can generally be classified into three components: bound water (T_21_), immobilized water (T_22_), and free water (T_23_).

As shown in Figure 1B, different “Paozhi” methods markedly affected the water distribution in GE. Fresh GE exhibited typical characteristics of high-moisture plant tissue, with water predominantly present in the free-water state [22]. Specifically, the proportion of T_23_ reached 97.93% (Figure 1C), whereas bound water (T_21_) and immobilized water (T_22_) accounted for only 0.05% and 2.02%, respectively. These results indicate that, in the unprocessed state, water in GE primarily existed in a free form within intact cell cavities and vacuoles, with minimal interaction with the surrounding matrix.

Processing treatments significantly altered the internal water distribution pattern of GE. In GE-stm, the proportion of T_23_ decreased sharply from 97.93% to 2.62%, while T_21_ and T_22_ increased markedly to 48.25% and 49.12%, respectively. According to [23], this transformation can be attributed to starch gelatinization and gel network formation induced by high-temperature steaming. During this process, starch granules absorb water and heat, undergo swelling, and lose their crystalline structure, gradually transitioning into an amorphous state. These granules subsequently interact and cross-link to form a continuous three-dimensional gel network. As a result, free water is either incorporated into the swollen biopolymeric matrix or bound to newly exposed hydrophilic groups, leading to its conversion into bound water, while a substantial portion becomes physically entrapped within the gel network as immobilized water. This interpretation is consistent with the film-like and massive structures observed in the SEM images. These findings are consistent with those of Xie et al. [24], who reported that steaming and water blanching damaged the cellular structure of GE, promoted starch aggregation and gelatinization, and shifted the T_2_ relaxation signals toward shorter relaxation times, indicating restricted water mobility. However, the relative distribution of the water fractions differed between the two studies. This discrepancy may be related to differences in thermal intensity, treatment endpoint, and subsequent drying conditions. In the present study, GE was steamed at 100 °C for 20 min and then hot-air dried to approximately 15% moisture, which may have further promoted water binding and physical entrapment within the gelatinized starch network. These comparisons indicate that both hydrothermal treatment and subsequent dehydration contribute to the final water distribution of processed GE.

In contrast, GE-ms exhibited a decrease in the proportion of T_23_ to 48.64%, accompanied by a significant increase in T_22_ from 2.02% to 47.17%. SEM observations further confirmed that moistening largely preserved the vascular bundle structure and overall microstructural integrity of GE. This relatively intact microporous architecture facilitated the penetration of external water into the tissue via osmotic driving forces, promoting interactions with starch and enhancing water retention within the granular substructure. Consequently, free water was partially converted into immobilized water, leading to the pronounced increase in T_22_ [23]. GE-wbf exhibited a water distribution pattern similar to that of GE-stm. Compared with fresh GE, the proportion of T_23_ decreased significantly to 10.33%, while the proportion of bound water increased markedly to 44.83%. This phenomenon is closely associated with the dense surface “crust” observed in SEM images. During bran-frying, high-temperature conditions promote rapid evaporation of free water, along with Maillard reactions and protein denaturation, resulting in the formation of a compact surface barrier [24]. Simultaneously, conformational changes in internal macromolecules may expose additional hydrophilic binding sites, thereby enhancing the matrix’s capacity to chemically bind the remaining water.

### 3.2. Chemical and Bioactive Component Analysis of GE

**Figure 2:**
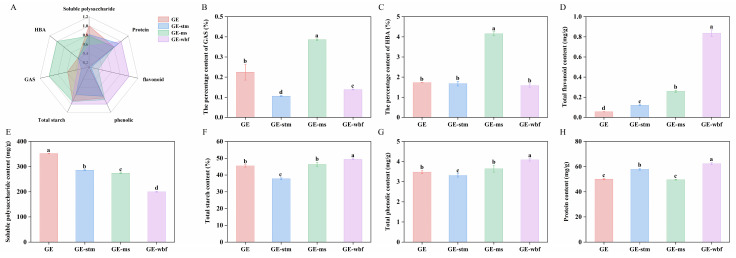
(**A**) Radar chart of seven active components in GE. (**B**) Gastrodin (GAS) content. (**C**) p-Hydroxybenzyl alcohol (HBA) content. (**D**) Total flavonoid content. (**E**) Soluble polysaccharide content. (**F**) Total starch content. (**G**) Total phenolic content. (**H**) Protein content. Different letters indicate significant differences (*p* < 0.05). GE: Fresh *Gastrodia elata* (GE), GE-stm: Steamed GE, GE-ms: moistened GE, GE-wbf: bran-fried GE.

Different processing methods induced significant alterations in the microstructure of GE, which not only affected water distribution but also influenced the composition and content of its bioactive constituents. In this study, seven key components were analyzed, including gastrodin (GAS), p-hydroxybenzyl alcohol (HBA), starch, soluble polysaccharides, total phenolics, total flavonoids, and protein. A radar plot was constructed using these components as variables (Figure 2A). The results revealed distinct compositional differences among processing treatments. GE-wbf exhibited the highest values for protein, total flavonoids, total phenolics, and starch. In contrast, GE-ms showed the highest levels of GAS and HBA, while fresh GE exhibited the highest soluble polysaccharide content. Overall, GE-stm displayed relatively low levels of most bioactive components. A more detailed quantitative analysis demonstrated that GE-ms contained the highest concentrations of GAS (0.38%) and HBA (4.16%) (Figure 2B,C), both significantly higher than those in the other processed groups. In contrast, GE-wbf exhibited the highest levels of total flavonoids (0.84%), protein (62.56 mg/g), starch (49.45 mg/g), and total phenolics (4.09 mg/g) (Figure 2D,F–H), all of which were significantly higher than those observed in the other treatments. Meanwhile, the soluble polysaccharide content (Figure 2E) was significantly higher in fresh GE than in all processed samples. Previous studies have demonstrated that processing methods such as steaming and drying can markedly alter both the composition and abundance of endogenous constituents in GE [25]. These changes are closely associated with processing-induced modifications of microstructure under high-temperature and high-moisture conditions. As a monobenzyl compound, HBA exhibits high permeability across the intestinal epithelium and can readily cross the blood–brain barrier, thereby contributing to its sedative and antiepileptic effects. However, HBA content has been reported to decrease with prolonged heating, likely due to thermal degradation into volatile products [26]. This mechanism may explain the reduced HBA levels observed in GE-stm and GE-wbf following high-temperature treatments.

Similarly, in fresh GE, endogenous β-glucosidase can promote the degradation of GAS, and thermal processing is typically employed to inactivate this enzyme [27]. Nevertheless, the GAS contents in GE-stm and GE-wbf remained lower than those in fresh GE. This may be attributed to the formation of a dense surface layer composed primarily of gelatinized starch under high-temperature conditions, which hinders the release and extraction of active compounds.

In contrast, GE-ms effectively preserved the native honeycomb-like cellular structure and well-defined vascular bundles, thereby maintaining an open and porous network. LF-NMR results further indicated that moistening significantly promoted water penetration into the tissue via osmotic driving forces, increasing the proportion of immobilized water from 2.02% to 47.17%. This enhanced hydration likely induced relaxation of the cell wall matrix, weakening its binding to low-molecular-weight compounds such as GAS and HBA and facilitating their release. Moreover, as shown in Figure 2G, no significant differences were observed between GE-ms and fresh GE in terms of total phenolics, starch, total flavonoids, or protein content. This suggests that the relatively mild conditions of moistening effectively minimized oxidation and degradation, thereby preserving the native composition of GE [5]. Although both GE-stm and GE-wbf involve high-temperature processing, notable differences were observed in their compositional profiles, particularly in total phenolics and starch content. This discrepancy is likely attributable to differences in thermal environments, with GE-stm subjected to high-temperature, high-moisture conditions, whereas GE-wbf undergoes dry-heat treatment. LF-NMR results further confirmed that the free-water content in GE-wbf decreased sharply from 97.93% to 10.33% without the introduction of exogenous moisture. This dehydration likely resulted in a concentration effect, thereby increasing the relative content of bioactive constituents on a mass basis. It should be noted that the Folin–Ciocalteu assay is based on the reduction of phosphomolybdic–phosphotungstic reagents and is therefore not entirely specific to phenolic compounds. Other reducing constituents co-extracted with ethanol, including reducing sugars and other redox-active molecules, may also contribute to the measured absorbance and potentially overestimate the apparent total phenolic content. Moreover, processing may alter both the abundance and extractability of these interfering substances, and differences in matrix effects among treatments cannot be completely excluded. The aluminum-complexation assay used for total flavonoid determination is less directly affected by reducing sugars; however, its response depends on the structural characteristics and aluminum-binding capacities of individual flavonoid subclasses. Other co-extracted compounds capable of complexing with aluminum ions or absorbing at 510 nm may also influence the results. Therefore, the total phenolic and total flavonoid contents reported in this study should be interpreted as comparative colorimetric estimates obtained under standardized extraction and reaction conditions rather than as absolute concentrations of structurally defined phenolic or flavonoid compounds.

In addition, the soluble polysaccharide content in fresh GE was significantly higher than that in all processed groups (Figure 2E). The reductions observed in GE-stm and GE-wbf may be attributed to the physical entrapment of polysaccharides within the network structure formed by starch gelatinization. In the case of GE-ms, although the microstructure remained relatively intact, prolonged moistening led to substantial water uptake and increased immobilized water, resulting in a dilution effect and a corresponding decrease in the relative soluble polysaccharide content.

### 3.3. Electronic Nose and Electronic Tongue Response Analysis

**Figure 3:**
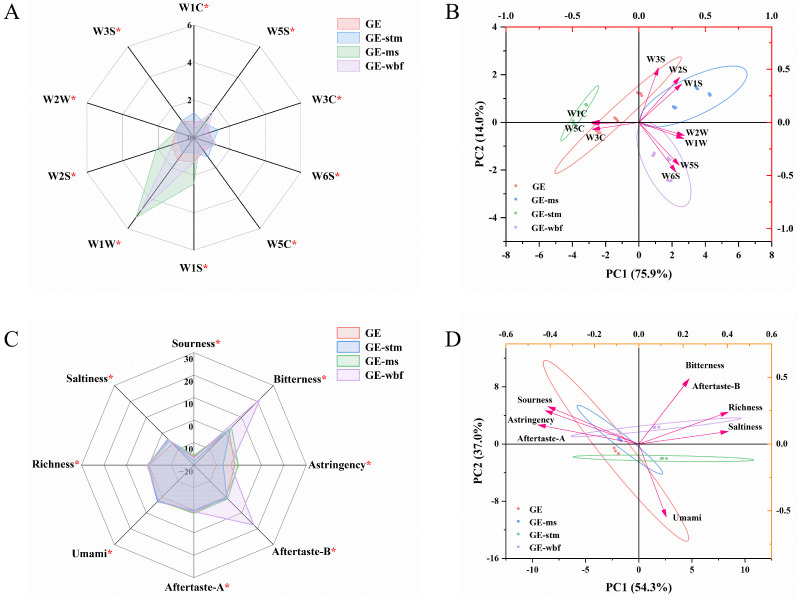
(**A**) Electronic nose radar plot. (**B**) Principal component analysis (PCA) of electronic nose data. (**C**) Electronic tongue radar plot. (**D**) Principal component analysis (PCA) of electronic tongue data. An asterisk (*) indicates a significant difference in the response of the corresponding electronic-nose sensor among the four sample groups (*p* < 0.05). GE: Fresh *Gastrodia elata* (GE), GE-stm: Steamed GE, GE-ms: moistened GE, GE-wbf: bran-fried GE.

The electronic nose, as a rapid and efficient analytical tool, has been widely applied for the detection of volatile compounds in food systems [28]. As shown in Figure 3A, significant differences were observed in the responses of all sensors among samples subjected to different processing treatments. Notably, pronounced variations were detected for W1W (sulfur-containing compounds), W5C (hydrocarbon aromatic compounds), W1C (aromatic compounds), and W1S (short-chain alkanes), indicating that “Paozhi” markedly influenced the volatile flavor characteristics of GE.

Specifically, moistening was more effective in preserving the original delicate aroma of GE, whereas steaming promoted the formation of aroma-related compounds associated with aromatic notes. In contrast, bran-frying significantly enhanced sensor responses related to sulfur-containing compounds, nitrogen-containing compounds, and other complex volatiles, thereby imparting a more intense roasted aroma and a richer flavor profile. These observations are consistent with the microstructural alterations and water redistribution patterns revealed by SEM and LF-NMR analyses, suggesting that “Paozhi” drives divergence in volatile flavor profiles by modulating tissue structure, water status, and associated chemical reactions.

Principal component analysis (PCA) further demonstrated that PC1 and PC2 accounted for 75.9% and 14.0% of the total variance, respectively, with a cumulative contribution of 89.9% (Figure 3B). This indicates that the two principal components effectively captured the key information of volatile compounds and clearly differentiated the samples according to processing treatments.

To further evaluate taste characteristics, an electronic tongue was employed to simulate human gustatory perception, including sourness, bitterness, umami, saltiness, and astringency. As shown in Figure 3C, significant differences were observed in all taste attributes among the different treatment groups, indicating that “Paozhi” not only altered volatile flavor profiles but also significantly affected non-volatile components.

GE-wbf exhibited significantly higher response values for bitterness and aftertaste-B compared with other groups, along with relatively strong responses in richness and saltiness. These results suggest that bran-frying markedly enhanced taste intensity and mouthfeel complexity, resulting in a fuller and more robust flavor profile. In contrast, the response values of GE-stm were generally lower than those of fresh GE, which is consistent with previous findings [15], indicating that unprocessed GE releases stronger odor-related compounds than steamed samples. Meanwhile, the taste profile of GE-ms remained largely similar to that of fresh GE, suggesting that moistening exerted minimal influence on overall taste characteristics. This behavior is likely associated with the preservation of microstructural integrity, as observed in SEM analysis. Further PCA (Figure 3D) showed that PC1 and PC2 explained 54.3% and 37.0% of the total variance, respectively, with a cumulative contribution of 91.3%, indicating effective discrimination among samples based on taste attributes. Overall, the electronic tongue results confirmed that different “Paozhi” methods significantly altered the taste characteristics of GE, with bran-frying enhancing bitterness and overall taste intensity, whereas moistening preserved a profile like that of fresh GE.

In summary, different “Paozhi” methods markedly influenced the overall flavor characteristics of GE. The GE-wbf group exhibited the strongest responses in both volatile (e.g., sulfur- and nitrogen-containing compounds) and taste-related attributes (e.g., bitterness, richness, and saltiness), indicating a more intense and complex flavor profile. In contrast, GE-stm enhanced aromatic and umami-related characteristics, whereas GE-ms maintained a flavor profile closely resembling that of fresh GE.

Although the electronic nose and electronic tongue effectively captured the overall sensory differences among samples, they do not provide detailed information on the underlying chemical composition. Therefore, HS-SPME-GC-MS was subsequently employed to identify volatile compounds, while UPLC-MS/MS was used to characterize differential non-volatile metabolites, to further elucidate the molecular basis of flavor formation.

### 3.4. Analysis of HS-SPME-GC-MS

**Figure 4:**
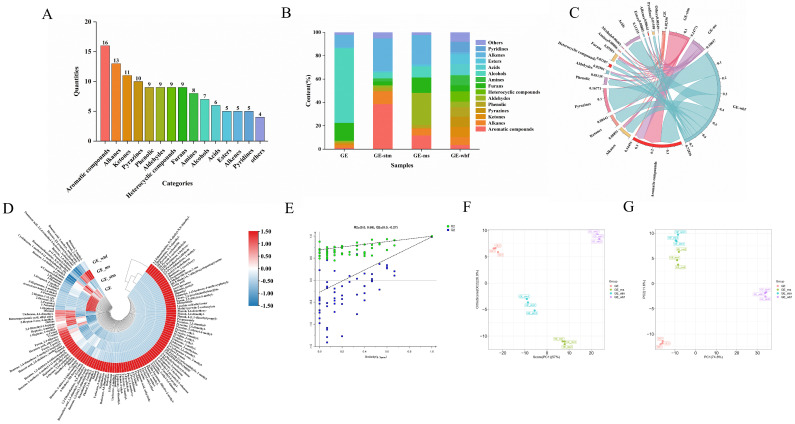
(**A**) Number of volatile compounds in different categories. (**B**) Relative content of volatile compounds in different categories. (**C**) Chord diagram of volatile compound categories in GE, GE-stm, GE-ms, and GE-wbf. (**D**) Heatmap of volatile compounds. (**E**) Permutation test of the OPLS-DA model. (**F**) OPLS-DA score plot. (**G**) PCA score plot. Filter criteria: VIP > 1 as well as *p* < 0.01. GE: Fresh *Gastrodia elata* (GE), GE-stm: Steamed GE, GE-ms: moistened GE, GE-wbf: bran-fried GE.

The complex chemical transformations induced by different “Paozhi” methods resulted in pronounced alterations in the flavor profile of GE, thereby significantly influencing sensory perception and potential consumer preference. In this study, a total of 125 volatile compounds were identified and classified into 14 categories, including aromatic compounds (16), alkanes (13), and ketones (11) (Figure 4A). As shown in Figure 4C and Table S1, substantial differences in the composition and relative abundance of major volatile compounds were observed among the treatment groups, particularly in aromatic compounds, ketones, and acids. Notably, the GE-wbf group exhibited the highest proportion of volatile organic compounds (72.83%), whereas the GE group showed a markedly lower proportion (2.35%). Aromatic compounds and their derivatives represent key contributors to the characteristic flavor and sensory quality of GE [29]. In the present study, aromatic compounds constituted the predominant class, accounting for 34.45% of total volatiles, with processed samples contributing the majority. This predominance is likely associated with heat-induced reactions during “Paozhi”, including Maillard reactions, hydrolysis or cleavage of phenolic and carbohydrate precursors, and the release of bound aroma precursors following cellular disruption. In addition, changes in water distribution and dehydration-induced concentration effects may have further facilitated the release and detection of low-molecular-weight aromatic compounds [30]. To further elucidate the variation patterns of volatile compounds, a clustered heatmap based on compound concentrations was constructed (Figure 4D). Aromatic compounds in GE primarily include monobenzyl compounds, polyaromatic glycosides, polyphenyl ethers, and polyphenyl compounds. Among these, gastrodin, a representative monobenzyl compound, serves as an important bioactive marker of GE [31]. In addition to imparting roasted, smoky, and caramel-like notes, aromatic compounds can interact synergistically with other volatile precursors, such as phenolics, thereby enhancing aroma complexity and layering [32]. Notably, Benzene, [(2-propenyloxy) methyl]-, a compound associated with a sweet aromatic note, was absent in both GE-stm and GE-wbf compared with GE and GE-ms. This absence may be attributed to the thermal instability of the allyl group (CH_2_=CH–CH_2_–), which is prone to cleavage or isomerization during high-temperature processing. In contrast, the content of Benzene, 1,3-bis(1,1-dimethylethyl)-, an aromatic compound with a slightly sweet note, was significantly higher in GE-wbf, contributing to a more pronounced roasted or woody aroma [33]. This increase is likely associated with the more intense heat transfer conditions during bran-frying.

In addition, alcohol accounted for 70.95% of the total alcohol content in fresh GE, which was significantly higher than in the processed groups. This observation is consistent with previous findings indicating that fresh or freeze-dried GE retains its native flavor profile, with alcohols as the dominant contributors [5]. Representative alcohols, such as 1-heptanol and 1-octanol, contribute fruity, floral, and green notes to fresh GE. Furthermore, alcohols can participate in Maillard reactions, and elevated temperatures accelerate their conversion into aromatic and furan compounds [34]. This mechanism may partially explain the observed increase in aromatic compounds during steaming and bran-frying, which shifts the flavor profile from delicate and sweet toward roasted and caramel-like characteristics.

Aldehydes, typically generated through lipid oxidation and degradation, play a crucial role in aroma formation due to their low odor thresholds [35]. In this study, nine aldehydes were identified, with GE-wbf exhibiting the highest aldehyde content (54.30%). Among these, decanal and nonanal were predominant, contributing fresh and citrus-like notes owing to their extremely low odor thresholds (0.0026 and 0.0031, respectively).

Ketones, on the other hand, are generally associated with milky, nutty, mushroom-like, and earthy aromas, providing a fundamental flavor base and enhancing overall complexity. The results showed that, concomitant with the decrease in alcohol content, ketone levels increased significantly following processing. This increase may be attributed to the formation of ketones via alcohol oxidation during thermal treatment [36]. Consequently, the elevated ketone content likely acted synergistically with aromatic compounds, aldehydes, and furans, thereby enhancing both flavor layering and overall sensory complexity.

Similar processing-dependent differentiation of volatile profiles has also been reported in GE. Ma et al. [27] combined electronic nose analysis with HS-SPME-GC-MS and demonstrated that different drying techniques generated distinct volatile compositions, particularly in aldehydes, alcohols, and hydrocarbons. Consistent with their findings, the present study showed that processing conditions were a major determinant of the volatile profile of GE. However, compared with conventional drying treatments, bran-frying produced a more pronounced enrichment of aromatic compounds, aldehydes, ketones, and thermally derived volatiles. This difference may be attributed to the dry-heat environment, rapid dehydration, and wheat-bran-mediated heat transfer, which collectively intensified lipid oxidation, Maillard reactions, and the conversion of endogenous flavor precursors.

Overall, GE-wbf and GE-stm exhibited richer flavor compositions and higher concentrations of volatile compounds, primarily due to the intensive thermal conditions that promoted extensive transformation of endogenous constituents. The enrichment of roasted, caramel-like, and citrus-like notes contributed to a more complex sensory profile and significantly altered the native flavor characteristics of GE. Therefore, the observed flavor shifts following processing are not solely attributable to changes in the total abundance of volatile compounds but more importantly to compositional transformations driven by high-temperature processing.

### 3.5. Analysis of UPLC-MS/MS

**Figure 5:**
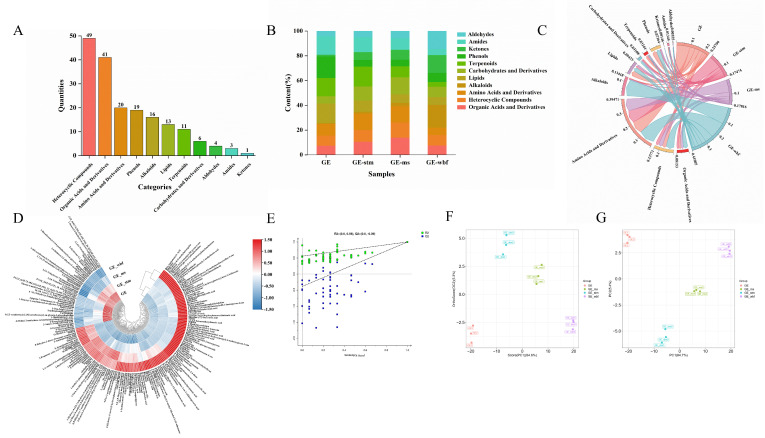
(**A**) Number of differential metabolites in different categories. (**B**) Relative content of differential metabolites in different categories. (**C**) Chord diagram of differential metabolite categories in GE, GE-stm, GE-ms, and GE-wbf. (**D**) Heatmap of differential metabolites. (**E**) Permutation test of the OPLS-DA model. (**F**) OPLS-DA score plot. (**G**) PCA score plot. Filter criteria: VIP > 1 as well as *p* < 0.01. GE: Fresh *Gastrodia elata* (GE), GE-stm: Steamed GE, GE-ms: moistened GE, GE-wbf: bran-fried GE.

Following GC-MS analysis of volatile compounds, liquid chromatography–tandem mass spectrometry (LC-MS/MS) was further employed to characterize differential metabolites among the samples, thereby providing deeper insight into the effects of “Paozhi” on the flavor formation of GE. In total, 1283 metabolites were identified. To enhance the reliability and statistical significance of the analysis, differential metabolites were screened using the criteria VIP > 1 and *p* < 0.01, resulting in the identification of 184 significant metabolites. These metabolites were primarily classified into heterocyclic compounds (49), organic acids and their derivatives (41), amino acids and their derivatives (20), phenolic compounds (19), and other categories (Figure 5A).

Consistent with the volatile compound analysis, the GE-wbf group exhibited a markedly higher proportion of differential metabolites compared with the other groups, reaching 41.01% (Figure 5B,C). Principal component analysis (PCA) (Figure 5G) showed that PC1 and PC2 together explained 90.1% of the total variance, indicating that these components captured the majority of variability among samples and effectively distinguished the treatment groups. The clear separation of data points further confirmed significant differences in metabolite profiles among the four groups.

Cluster analysis of the quantified differential metabolites further revealed distinct variation patterns (Figure 5C and Table S2). Amino acids and their derivatives represented the largest proportion (39.47%) and exhibited significant variation among treatments. Notably, L-theanine, a known flavor-enhancing compound, has been reported to promote the formation of 2,5-dimethylpyrazine [37]. Its abundance increased by 96.21% in the GE-wbf group compared with the GE group, potentially contributing to the characteristic roasted peanut-like flavor of GE-wbf.

In contrast, DL-glutamic acid, an important umami-related amino acid, decreased by 53.59% in GE-wbf. This reduction may be attributed to its dehydration and oxidation into L-pyroglutamic acid under high-temperature conditions [38]. Heterocyclic compounds also contributed substantially to flavor formation, with a higher proportion observed in GE-wbf. These compounds are typically associated with roasted and fruity aroma characteristics generated during thermal processing, fermentation, and drying. For example, the levels of 4-hydroxyacetophenone and ethyl eugenol increased by 97.50% and 89.29%, respectively, in GE-wbf, which may impart fruity-sweet and vanilla-like notes.

Lipids also play an important role in flavor development. Although they possess relatively weak intrinsic sensory properties, they serve as key precursors of volatile compounds such as aldehydes and ketones, thereby influencing flavor both directly and indirectly [39]. Compared with fresh GE, the processed groups exhibited an overall reduction of 51.40% in lipid content. Meanwhile, aldehydes and ketones accounted for 98.9% and 98.34% of volatile compounds in the processed samples, respectively. This phenomenon is likely attributed to lipid oxidation and degradation during processing, which generate volatile compounds such as aldehydes, alcohols, and methyl ketones [40]. Furthermore, the alkaloid content in GE-wbf increased significantly. As alkaloids are typically associated with bitterness, this finding is consistent with the elevated bitterness response observed in the electronic tongue analysis, further supporting the relationship between metabolite composition and sensory perception.

In summary, “Paozhi” significantly reshaped the metabolite profile of GE. Variations in amino acids and their derivatives, heterocyclic compounds, lipids, and alkaloids collectively contributed to the distinct flavor characteristics of the processed samples. These compositional changes not only enhanced sweet, fruity, and vanilla-like notes, but also intensified umami and bitterness, thereby enriching the overall flavor complexity of GE.

### 3.6. Correlation Analysis Between E-Nose, E-Tongue, Volatile Compounds, and Differential Metabolites in GE

**Figure 6:**
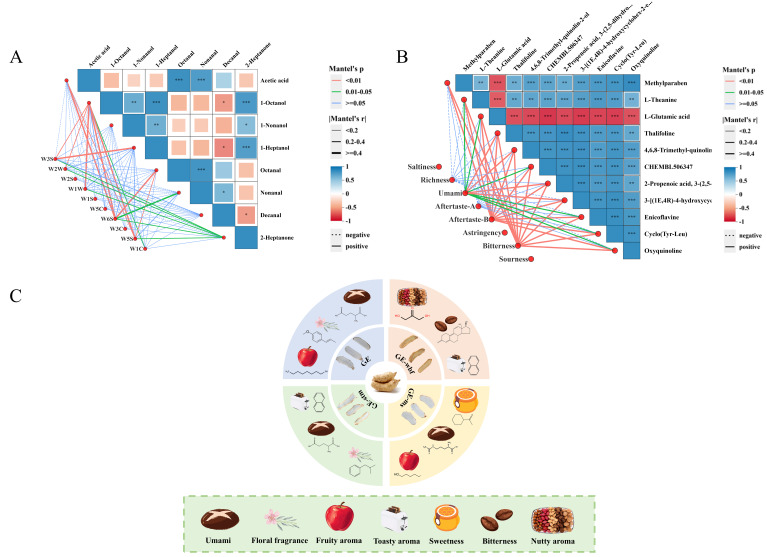
(**A**) Correlation heatmap between electronic nose sensor responses and key volatile compounds. (**B**) Correlation heatmap between electronic tongue responses and key differential metabolites. (**C**) Flavor wheel. GE and CON-GE: Fresh *Gastrodia elata* (GE), GE-stm: Steamed GE, GE-ms: moistened GE, GE-wbf: bran-fried GE. Asterisks indicate the significance levels of the correlations: * *p* < 0.05, ** *p* < 0.01, and *** *p* < 0.001. Some metabolite names are abbreviated with ellipses for clarity; their complete names are provided in Table S2.

The Mantel test, a matrix-based correlation analysis method, was employed to quantify the strength and significance of associations among electronic nose (E-nose) and electronic tongue (E-tongue) sensor responses, key volatile compounds, and differential metabolites. This approach enabled a comprehensive evaluation of the systematic effects of “Paozhi” on the flavor-related chemical composition and sensory characteristics of GE. A correlation network was constructed to examine the relationships between E-nose sensor responses and selected key volatile compounds (Figure 6A). The results showed that the W3S, W2W, W1W, W6S, and W5S sensors were significantly correlated with compounds such as acetic acid, 1-octanol, 1-heptanol, and octanal. Notably, the signal intensity of the W6S sensor exhibited a significant positive correlation with 1-octanol content, suggesting that alcohols contribute positively to desirable aroma attributes, including fresh, fruity, and floral notes. However, following “Paozhi”, the contents of alcohols and aldehydes decreased significantly across all processed groups, indicating that processing-induced changes in microstructure and water distribution may indirectly influence flavor formation. In addition, the acetic acid content in the GE-wbf group was markedly higher than in other groups, while the aroma-related W1C and W3C sensors showed significant negative correlations with acetic acid. This suggests that elevated acetic acid levels may adversely affect the overall aroma quality of GE-wbf. It is also noteworthy that certain aromatic compounds, such as 1-octanol, octanal, and 1-nonanol, were not effectively detected by the W1C and W3C sensors. This phenomenon may be attributed to competitive adsorption or occupation of sensor active sites by high-affinity compounds, which can hinder the detection of specific volatiles and result in attenuated or masked sensor responses [41]. Further correlation analysis between E-tongue responses and key differential metabolites revealed that bran-frying significantly increased the levels of alkaloids, including oxyquinoline, 4,6,8-trimethyl-quinolin-2-ol, and thalifoline. As shown in Figure 6B, aftertaste-B exhibited a significant positive correlation with these alkaloids and their derivatives, indicating that high-temperature, low-moisture conditions play a critical role in enhancing bitterness and overall taste intensity. Consistently, umami-related amino acids and their derivatives, such as L-glutamic acid and L-theanine, were positively associated with enhanced umami responses detected by the electronic tongue, thereby contributing to flavor enhancement. To further visualize the flavor characteristics of GE processed by different “Paozhi” methods, a flavor wheel was constructed (Figure 6C) by integrating electronic nose and electronic tongue data with key volatile compounds and differential metabolites. The GE-wbf group was characterized by pronounced toasty and nutty aromas, along with increased bitterness. These attributes are likely associated with the formation of sulfur-containing compounds, alkaloids, and heterocyclic compounds under high-temperature, low-moisture conditions, resulting in a rich and robust flavor profile. In contrast, GE-stm was primarily characterized by enhanced umami, floral, and mildly roasted notes, which may be attributed to microstructural alterations and water redistribution under high-temperature, high-moisture conditions that promote the release and transformation of aroma compounds. Meanwhile, GE-ms preserved the native microstructure to the greatest extent and exhibited a flavor profile closely resembling that of fresh GE, with only minor changes.

## 4. Conclusions

This study systematically compared the effects of three traditional “Paozhi” methods, steaming, moistening, and bran-frying, on the microstructure, water distribution, bioactive constituents, and flavor characteristics of *Gastrodia elata* (GE). The results demonstrated that the quality variations induced by different processing methods cannot be attributed to changes in a single factor but rather arise from the synergistic effects of tissue structure remodeling, water redistribution, and metabolite transformation. Moistening caused minimal disruption to the native tissue architecture of GE, with the proportion of free water decreasing from 97.93% to 48.64%. Under these conditions, the contents of gastrodin (GAS) and p-hydroxybenzyl alcohol (HBA) reached 0.38% and 4.16%, respectively, representing the highest levels among all treatment groups. Moreover, the overall flavor profile of GE-ms remained closest to that of fresh GE, indicating that moistening effectively preserves the intrinsic quality attributes of GE. In contrast, bran-frying induced pronounced dehydration and structural densification, with the proportion of free water decreasing to 10.33%. This treatment resulted in a substantial increase in volatile organic compounds (72.83%) and differential metabolites (41.01%), accompanied by elevated levels of total phenolics, total flavonoids, protein, and starch. These compositional changes contributed to the development of distinctive roasted and nutty flavor characteristics. Steaming exhibited intermediate effects between moistening and bran-frying. The proportion of free water further decreased to 2.62%, and the high-temperature, high-moisture conditions significantly altered both tissue structure and water distribution. Consequently, the flavor profile shifted from the original delicate aroma toward enhanced umami, floral, and mildly roasted notes. Overall, different “Paozhi” methods modulate the sensory characteristics of GE by regulating its structural matrix and metabolite composition. From an integrated perspective encompassing processing conditions, structural transformation, compositional variation, and flavor formation, this study elucidates the intrinsic mechanisms underlying quality evolution in GE. These findings not only provide a scientific basis for interpreting traditional processing practices but also offer valuable guidance for the precise processing and quality control of GE decoction pieces and medicine–food homology (MFH) products.

## Data Availability

The original contributions presented in this study are included in the article/supplementary material. Further inquiries can be directed to the corresponding author.

## Supplementary Materials

The following are available online at https://www.mdpi.com/article/10.3390/foods15152704/s1, Figure S1. Representative HPLC chromatograms of gastrodin and p-hydroxybenzyl alcohol in fresh and processed *Gastrodia elata*. (A) GE, (B) GE-stm, (C) GE-ms, and (D) GE-wbf. Peaks 1 and 2 correspond to gastrodin and p-hydroxybenzyl alcohol, respectively. GE, fresh *Gastrodia elata*; GE-stm, steamed *Gastrodia elata*; GE-ms, moistened *Gastrodia elata*; GE-wbf, bran-fried *Gastrodia elata*. Table S1. Normalized peak areas of volatile compounds screened using VIP > 1 and *p* < 0.05, together with their odor threshold values and relative odor activity values (ROAVs) in each sample group. Odor threshold values were obtained from *Compilations of Odour Threshold Values in Air, Water and Other Media* (Second Edition). Normalized peak areas are dimensionless and represent relative abundance rather than absolute concentration. Table S2. Normalized peak areas of differential non-volatile metabolites detected by UPLC-MS/MS and screened using VIP > 1 and *p* < 0.05 in GE, GE-stm, GE-ms, and GE-wbf. The normalized peak areas are dimensionless and represent relative abundance rather than absolute concentration. Thank you for pointing this out.

## Abbreviations

The following abbreviations are used in this manuscript:

GE	*Gastrodia elata* Bl
GE-stm	Steamed *Gastrodia elata* Bl
GE-ms	Moistened *Gastrodia elata* Bl
GE-wbf	bran-fried *Gastrodia elata* Bl
