# Supplementary material for: Changes in the Quality Characteristics of Gastrodia Elata During Traditional Processing: Insights from Multi-Omics Integration
Source: Foods. 2026 Jul 31;15(15):2704. doi: 10.3390/foods15152704 (PMC13463839; doi:10.3390/foods15152704)
Supplement: Supplementary file 1 [file foods-15-02704-s001.zip › foods-4410450-supplementary.pdf]

Table S1 Normalized peak areas of volatile compounds screened using VIP > 1 and $p < 0.05$, together with their odor threshold values and relative odor activity values (ROAVs) in each sample group. Odor threshold values were obtained from Compilations of Odour Threshold Values in Air, Water and Other Media (Second Edition). Normalized peak areas are dimensionless and represent relative abundance rather than absolute concentration.

Items	Threshold	GE	ROAV	GE-stm	ROAV	GE-ms	ROAV	GE-wbf	ROAV
Benzene,1,2-dichloro-4-(chloromethyl)-		0		8.73 ± 13.98^{c}		82.22 ± 14.78b		219.61 ± 18.55a	
Benzene,1,3-bis(1,1-dimethylethyl)-		3997.14 ± 1046.17^{b}		6531.37 ± 670.28^{b}		9178.41 ± 1750.19^{b}		35997.39 ± 10593.85^{a}	
Benzene, 1,4-dichloro-		126.02 ± 43.95^{ab}		82.01 ± 92.09^{ab}		173.71 ± 81.96^{ab}		0	
Benzene,1,4-dichloro-2-methyl-		128.60 ± 44.60^{a}		150.39 ± 43.86^{a}		90.1326 ± 156.11^{a}		0	
Benzene, 1,4-dimethoxy-2-methyl-5-isopropyl-		300.46 ± 35.83^{c}		81.04 ± 23.55^{d}		556.42 ± 26.91b		1101.35 ± 39.65^{a}	
Benzene,1-chloro-3-(chloromethyl)-		0		53.49 ± 13.31^{b}		51.8124 ± 46.59^{b}		241.61 ± 4.24^{a}	
Benzene, 2-methoxy-4-methyl-1-(1-methylethyl)-		770.65 ± 89.55546^{b}		382.36 ± 113.99^{c}		1031.04 ± 216.43^{b}		2978.94 ± 293.50^{a}	
Benzene, [(2-propenyloxy)methyl]-		148.63 ± 79.96		0		156.87 ± 271.72		0	
Benzene, pentyl-		0		0		0		660.97 ± 59.24a	
Benzenemethanamine, N, N-dimethyl-		78.66 ± 68.64^{b}		787.22 ± 201.71^{a}		0		0	
Benzenepropanoic acid, ethyl ester		8.27 ± 8.09^{b}		0		145.3904 ± 24.1723^{a}		126.20 ± 16.32^{a}	
Naphthalene	0.45	519.19 ± 79.96^{b}	0.23	666.47 ± 137.90^{b}	0.23	648.76 ± 103.89^{b}	0.07	2200.31 ± 95.20^{a}	<0.10
o-Cymene		0		541.20 ± 474.08b		444.52 ± 769.94b		2287.18 ± 149.08a	
p-(1-Propenyl)-toluene		122.04 ± 2.73		110.46 ± 101.02		0		0	
p-Cresol		5191.85 ± 394.36^{c}		353599.06 ± 67005.72^{a}		162036.38 ± 3904.21^{b}		354227.55 ± 26432.13^{a}	
p-Cymene		1846.59 ± 90.76^{a}		974.91 ± 847.75^{ab}		990.62 ± 867.37^{ab}		0	
**Aromatic compounds**		**13241.24 ± 1324.21^{c}**		**363971.64 ± 67327.66^{a}**		**175590.08 ± 3839.36^{b}**		**400045.13 ± 35005.75^{a}**	
5-Ethyldecane		0		27.93 ± 48.38^{b}		0		947.66 ± 230.49^{a}	
Decane		5050.33 ± 1218.19^{c}		17970.71 ± 4860.50^{b}		14535.94 ± 2609.62^{b}		44449.98 ± 5769.04^{a}	
Dodecane	0.77	0	0	4187.68 ± 918.47^{b}	0.87	1417.31 ± 2454.85^{b}	<0.10	112164.43 ± 14046.66^{a}	0.61
Dodecane, 4,6-dimethyl-		0		558.08 ± 277.44^{b}		516.26 ± 92.84^{b}		8065.17 ± 2541.92^{a}	
Heptane, 4-methyl-		288.51 ± 57.29^{c}		1051.84 ± 380.79^{b}		1718.19 ± 39.51^{a}		1201.86 ± 79.59^{b}	
Nonane, 3-methyl-5-propyl-		0		0		0		478.68 ± 448.66	

Octane	8	734.56 ± 151.92^{b}	<0.10	2237.66 ± 734.55^{a}	<0.10	2468.83 ± 175.88^{a}	<0.10	2248.04 ± 167.40^{a}	<0.10
Tridecane, 3-methyl-		0		0		0		9527.27 ± 603.76	
Tridecane, 5-methyl-		0		10.67 ± 18.48		0		2296.02 ± 1597.61	
Undecane, 3,8-dimethyl-		36.85 ± 63.84^{b}		0		87.31 ± 77.94^{b}		2703.16 ± 1709.86^{a}	
Undecane, 3-methyl-		240.46 ± 11.21^{b}		356.29 ± 7.92^{b}		462.86 ± 89.15^{b}		4898.27 ± 983.72^{a}	
Undecane, 3-methylene-		0		0		0		811.72 ± 50.76	
Undecane, 4,6-dimethyl-		0		68.50 ± 64.60^{a}		1016.13 ± 1680.84^{a}		1065.97 ± 138.51^{a}	
**Alkanes**		**6356.46 ± 21362.41^{c}**		**26473.13 ± 17131.71^{b}**		**22227.05 ± 35123.72^{bc}**		**190858.28 ± 3016630.98^{a}**	
1-(5-Methyl-2-pyrazinyl)-1-ethanone		0		0		0		1507.09 ± 126.84	
2-Butanone, 1-(acetyloxy)-		0		0		0		21264.31 ± 767.38	
2-Cyclopenten-1-one, 2-methyl-		0		0		0		1868.98 ± 87.43	
2-Heptanone	0.023	369.04 ± 41.80	3.17	0	0	84.48 ± 146.32	0.19	0	0
2-Hydroxy-3-pentanone	2.5	0	0	0	0	0	0	864.21 ± 51.68	<0.10
2-Octanone		636.49 ± 87.88		0		122.06 ± 106.09		0	
2-Propanone, 1-hydroxy-		0		0		0		200994.81 ± 72420.08	
3-Heptanone, 5-methyl-		1089.92 ± 76.87		0		0		0	
3-Pentanone	3	1257.97 ± 167.04	<0.10	0	0	0	0	0	0
5-Hepten-2-one, 6-methyl-		529.87 ± 20.59^{c}		398.11 ± 30.58^{d}		1347.95 ± 55.02^{a}		1030.73 ± 132.47^{b}	
Ethanone, 1-(3-hydroxy-4-methoxyphenyl)-		0		0		0		81.18 ± 9.21	
**Ketones**		**3887.25 ± 335.64^{b}**		**404.71 ± 30.58^{b}**		**1560.43 ± 198.79^{b}**		**227613.97 ± 71452.38^{a}**	
Pyrazine		0		31.28 ± 27.10		0		49252.10 ± 1620.19	
Pyrazine, 2,3-dimethyl-		0		0		0		21663.02 ± 234.68	
Pyrazine, 2,5-dimethyl-		0		0		0		57643.68 ± 1028.58	
Pyrazine, 2,6-dimethyl-		0		0		0		97169.69 ± 932.07	
Pyrazine, 2-ethenyl-6-methyl-		0		0		0		3411.43 ± 147.55	

Pyrazine, 2-ethyl-3,5-dimethyl-		0		0		0		5898.41 ± 297.40	
Pyrazine, ethyl-		0		0		0		23515.40 ± 1050.16	
Pyrazine, methyl-		47.97 ± 41.76^{b}		23.11 ± 20.06^{b}		44.09 ± 2.42^{b}		159433.06 ± 1317.86^{a}	
Pyrazine, trimethyl-		0		0		0		40897.74 ± 1105.49a	
Acetylpyrazine	0.06	0	0	0	0	0	0	4809.17 ± 511.95	0.33
**Pyrazines**		**54.14 ± 41.39^{b}**		**60.12 ± 25.89^{b}**		**50.03 ± 2.42^{b}**		**463693.75 ± 4804.60^{a}**	
Creosol		0		2685.74 ± 703.33^{b}		1154.16 ± 79.99^{c}		3582.15 ± 312.56^{a}	
Phenol		1979.90 ± 689.65^{b}		1467.83 ± 257.75^{b}		4805.09 ± 316.17^{b}		55847.71 ± 4523.53^{a}	
Phenol, 2,4-dimethyl-		0		0		35.72 ± 61.88		347.72 ± 21.85	
Phenol, 2,6-dimethoxy-		0		0		0		2514.54 ± 244.20	
Phenol, 2-methoxy-		7.86 ± 7.28^{b}		0		167.21 ± 20.93^{b}		61031.55 ± 3203.39^{a}	
Phenol, 2-methyl-		0		0		0		4809.25 ± 287.06	
Phenol, 3,4-dimethyl-		0		0		0		179.42 ± 58.98a	
Phenol, 4-(1,1-dimethylpropyl)-		0		0		0		660.99 ± 80.74	
Phenol, 4-ethyl-		0		0		17.27 ± 15.04		705.31 ± 61.87	
**Phenolic**		**1992.61 ± 692.06^{b}**		**4158.20 ± 960.78^{b}**		**6182.78 ± 368.68^{b}**		**129678.68 ± 8330.27^{a}**	
2-Decenal, (E)-		160.81 ± 25.69		0		0		0	
3-Furaldehyde		0		20.32 ± 35.20		0		3817.20 ± 128.85	
Benzaldehyde, 3-methyl-		0		62.55 ± 12.95^{ab}		121.91 ± 41.20^{ab}		427.70 ± 374.83^{a}	
Butanal, 2-methyl-		148.25 ± 14.90^{c}		127.18 ± 11.34^{c}		8060.44 ± 1407.43^{a}		1727.97 ± 91.93^{b}	
Butanal, 3-methyl-		20.07 ± 34.76^{b}		135.27 ± 6.37^{b}		13156.39 ± 2181.19^{a}		1890.60 ± 132.12^{b}	
Decanal	0.0026	0	0	378.76 ± 70.50^{c}	23.48	1388.45 ± 64.59^{a}	27.71	1256.60 ± 20.97^{b}	2.02
Methional		0		129.73 ± 20.8^{a}		189.65 ± 327.35^{a}		5227.54 ± 7633.07^{a}	
Nonanal	0.0031	0	0	0	0	5973.45 ± 5255.07^{b}	100	20262.19 ± 1219.60^{a}	27.42
Octanal	0.0008	404.43 ± 62.36^{c}	100	496.28 ± 10.58^{bc}	100	622.99 ± 81.01^{b}	40.41	2947.83 ± 129.56^{a}	15.46
**Aldehydes**		**737.31 ± 75.20^{c}**		**1351.88 ± 111.25^{c}**		**29514.87 ± 2611.2^{b}**		**37558.55 ± 6616.24^{a}**	

5H-5-Methyl-6,7-dihydrocyclopentapyrazine	0	0	0	2865.30 ± 155.77
Methyl pyrrole-2-carboxylate	0	0		332.87 ± 32.79
Propanoic acid, anhydride	0	22.75 ± 21.10	0	10261.39 ± 10638.46
3,4-(Methylenedioxy)toluene	0	0	0	105.59 ± 12.65
2,5-Pyrrolidinedione, 1-methyl-	0	0	0	505.44 ± 162.97
2-Pyrrolidinone	0	0	4.79 ± 8.30	1713.76 ± 464.81
1H-Pyrrole-2-carbonitrile	0	0	0	141.59 ± 15.13
1H-Pyrrole-2-carboxaldehyde	0	0	0	38143.92 ± 4546.83
1H-Pyrrole-2-carboxaldehyde, 1-methyl-	0	0	0	6373.64 ± 80.69
**Heterocyclic compounds**	**0**	**28.26 ± 20.74[b]**	**10.51 ± 7.92[b]**	**60443.52 ± 14776.94[a]**
2,5-Furandicarboxaldehyde	0	0	0	338.34 ± 38.79
2-Acetyl-5-methylfuran	0	0	0	262.28 ± 16.01
3(2H)-Furanone, dihydro-2-methyl-	0	0	0	5112.05 ± 53.09
4-Methoxy-5H-furan-2-one	0	24.30 ± 1.42	0	160.00 ± 51.40
4-Methyl-5H-furan-2-one	0	0	0	9777.83 ± 1556.91
Furan, 2,2-[oxybis(methylene)]bis-	0	0	0	1057.03 ± 89.30
Furan, 2,3-dihydro-	0	3.25 ± 5.64[c]	519.91 ± 110.44[b]	987.16 ± 55.54[a]
Furan, 2,3-dihydro-5-methyl-	0	0	0	2968.29 ± 577.00
Furan, 2-pentyl-	26244.91 ± 578.60[b]	4975.82 ± 942.62[c]	31129.75 ± 5895.38[b]	70842.54 ± 10354.11[a]
**Furans**	**26249.53 ± 578.60[b]**	**5007.13 ± 939.15[c]**	**31653.62 ± 5837.48[b]**	**91167.21 ± 9444.96[a]**
1,2-Ethanediamine, N,N-diethyl-N,N-dimethyl-	0	0	0	1541.97 ± 32.60
1,2-Ethanediamine, N,N-dimethyl-	0	634.32 ± 549.62	0	2004.19 ± 76.29
1-Butanamine, N,N-dimethyl-	0	0	0	236.13 ± 41.61
2-Propenamide	0	0	0	749.00 ± 190.85
Acetamide	0	0	0	6498.60 ± 2329.47
Glutarimide	0	0	0	420.98 ± 97.08

2-OXOPROPIONAMIDE		0		0		0		11062.87 ± 2057.08	
Propanamide		0		0		0		1335.01 ± 421.70	
**Amines**		**0**		**638.50 ± 549.24**		**0**		**22306.81 ± 4820.64**	
1-Heptanol	0.023	2105.96 ± 291.01	18.11	0	0	222.95 ± 386.16	0.50	0	0
1-Nonanol 1-	0.018	1383.06 ± 1282.94^{a}	15.20	102.04 ± 7.67^{b}	0.91	222.75 ± 221.96^{b}	0.64	0	0
**Naphthalene**nol	0.1258	3565.37 ± 710.38^{a}	5.60	107.95 ± 186.99^{c}	0.13	1011.06 ± 121.27^{b}	0.41	0	0
1-Propanol	0.24	67.40 ± 10.49	< 0.1	0	0	0	0	0	0
1-Propanol, 2-methyl-		1652.30 ± 519.32		0		303.98 ± 290.17		0	
a-Terpineol		151.49 ± 12.93^{c}		466.34 ± 99.26^{b}		182.54 ± 11.40^{c}		1320.75 ± 224.25^{a}	
2-Hepten-1-ol, (Z)-		724.87 ± 112.43		0		0		0	
**Alcohols**		**9650.70 ± 2491.68^{a}**		**679.42 ± 234.03^{b}**		**1945.50 ± 674.95^{b}**		**1324.71 ± 224.25^{b}**	
Benzoic acid, 2,5-dichloro-, methyl ester		13.49 ± 23.37^{b}		0		69.04 ± 55.36^{ab}		175.49 ± 98.69^{a}	
Benzoic acid, 2-methyl-, methyl ester		108.96 ± 6.36^{b}		43.91 ± 14.08^{c}		365.59 ± 33.39^{a}		0	
Heptanoic acid, ethyl ester		107.22 ± 14.39^{bc}		0		302.98 ± 13.14^{b}		1380.87 ± 315.15^{a}	
Linoleic acid ethyl ester		0		0		0		305.89 ± 36.07a	
Pentanoic acid, 2,2,4-trimethyl-3-hydroxy-, isobutyl ester		0		281.83 ± 71.60^{a}		154.76 ± 27.46^{b}		271.60 ± 63.26^{a}	
Acetic acid	0.013	0	0	0	0	0	0	309830.021 ± 84430.60a	100
**Acids**		**503.05 ± 54.92^{b}**		**201.31 ± 15.30^{b}**		**1020.78 ± 118.74^{b}**		**311693.61 ± 84254.21^{a}**	
Butanedioic acid, 2,3-dimethyl-, dimethyl ester		55.40 ± 3.12^{d}		104.92 ± 13.78^{c}		243.04 ± 9.02^{b}		986.56 ± 43.68^{a}	
Decanoic acid, ethyl ester		51.00 ± 22.95^{c}		0		170.74 ± 30.29^{b}		280.87 ± 37.92^{a}	
Furfuryl ethyl ether		0		8.64 ± 14.97		0		20578.04 ± 1896.74	
Furfuryl formate		0		0		0		876.92 ± 19.98	
Dimethyl phthalate		62.06 ± 8.93^{c}		222.02 ± 59.01^{b}		243.68 ± 41.91^{b}		495.47 ± 62.36^{a}	
**Esters**		**169.79 ± 28.14^{b}**		**337.35 ± 58.44^{b}**		**658.79 ± 78.54^{b}**		**23217.89 ± 1950.52^{a}**	

1-Heptene, 2-methyl-		0		144.19 ± 49.01^{c}		298.20 ± 19.83^{a}		205.89 ± 6.18^{b}	
2,4-Dimethyl-1-heptene		1063.09 ± 264.19^{c}		3488.58 ± 1261.23^{b}		5533.19 ± 159.66^{a}		3326.87 ± 328.27^{b}	
trans,trans-nona-2,4-dienol		80.22 ± 75.05		0		0		0	
4-Octene, 2,3,6-trimethyl-		19.55 ± 33.86^{b}		0		71.25 ± 123.41^{b}		284.44 ± 44.73^{a}	
Cyclohexene, 1-methyl-4-(1-methylethenyl)-, (S)-		975.52 ± 41.51^{b}		1186.35 ± 75.37^{a}		1041.66 ± 164.07^{ab}		0	
**Alkenes**		**2139.49 ± 295.84^{c}**		**4820.45 ± 1360.3^{b}**		**6945.42 ± 394.43^{a}**		**3818.53 ± 369.99^{b}**	
2(1H)-Pyridinone, 6-hydroxy-		0		0		0		3348.53 ± 652.61a	
3-Pyridinol		11.494 ± 19.90		0		0		3979.57 ± 164.10	
Pyridine	0.32	0	0	55.78 ± 14.12	<0.10	0	0	34011.98 ± 2223.98	0.44
Pyridine, 2-ethyl-		0		0		0		1091.88 ± 45.82	
Pyridine, 3-ethyl-		0		0		0		588.60 ± 33.32	
**Pyridines**		**14.57 ± 19.52^{b}**		**58.42 ± 14.12^{b}**		**0**		**43020.59 ± 1520.40^{a}**	
Undecane,5-methyl-		0		319.86 ± 76.61^{b}		174.14 ± 301.62^{b}		3072.19 ± 476.80^{a}	
4-Cyanocyclohexene		147.44 ± 15.13^{a}		24.97 ± 3.03^{bc}		54.06 ± 46.83^{b}		0	
Dimethyl		0		0		0		483.071 ± 187.66609	
**Others**		**149.42 ± 15.13^{b}**		**346.16 ± 76.62^{b}**		**230.19 ± 254.80^{b}**		**6164.07 ± 421.93^{a}**	

Results are presented as mean ± standard deviation. Columns with different letters indicate a significant difference ($p < 0.05$) according to Duncan's multiple range test. The different superscript letters (a-d) indicate a significant difference at the 0.05 significance level ($p < 0.05$).

TableS2 Normalized peak areas of differential non-volatile metabolites detected by UPLC-MS/MS and screened using VIP > 1 and $p < 0.05$ in GE, GE-stm, GE-ms, and GE-wbf. The normalized peak areas are dimensionless and represent relative abundance rather than absolute concentration.

Volatile flavor compound	RT	GE	GE-stm	GE-ms	GE-wbf
**4-Hydroxyphenylglycolic acid**	**70.9**	**1773191.25 ± 78872.5[a]**	**4325096.79 ± 1083723.08[b]**	**6970013.15 ± 1456798.85[c]**	**20202193.35 ± 18155415.24[d]**
3,7,11,15-tetramethyl-6,10,14-hexadecatrienyl acetate	369.4	5136287.06 ± 1652001.76	4256411.43 ± 125285.32	2426674.18 ± 559308.74	862541.97 ± 142924.44
3-[(E)-prop-1-enyl]pentanedioic acid	86.2	411542.59 ± 13282.4	4880340.87 ± 315213.91	4787059.64 ± 3259531.28	49738483.14 ± 1405867.19
Diphenylacetic Acid	119.9	4020225.41 ± 309273.83	52007478.38 ± 5108934.63	70541887 ± 2835031.74	96584511.9 ± 933308.83
Floionolic acid	312.2	102443.66 ± 169351.35	33656.73 ± 4417.82	650448.11 ± 85250.28	7194679.76 ± 333297.7
Diamyl phthalate	123.2	13500617.2 ± 788547.9	9536263.83 ± 1698676.56	892547.48 ± 184307.12	596647.29 ± 55860.47
3-Hydroxy-4-(2,6,7-trihydroxy-6-methylheptan-2-yl)benzoic acid	258.2	6564591.38 ± 292350.95	18725866.19 ± 1569278.22	25234428.36 ± 1457631.72	24408906.9 ± 2008814.84
**2-Propenoic acid, 3-(2,5-dihydroxyphenyl)-**	**209.2**	**1446519.78 ± 102381.48[d]**	**1840542.29 ± 10980.27[c]**	**3097957.65 ± 134780.18[b]**	**4057190.89 ± 229126.75[a]**
2,6-Dimethoxybenzoic Acid	196.7	916530.33 ± 734378.5	3722517.96 ± 196100.11	5529122.73 ± 609697.03	29610772.03 ± 74314.11
2-(3-Hexyl-4-methyl-2,5-dioxopyrrol-1-yl)-3-hydroxybutanoic acid	259.8	4678486.58 ± 277974.64	10152512.19 ± 725143.9	17801705.13 ± 1221731.4	16028487.4 ± 736017.38
3-Methylglutaric acid	42.9	223460489.26 ± 14419918.1	110743269.8 ± 2891872.66	75014258.49 ± 5427611.45	83743346.19 ± 1872849.45
Me ether-(??)-Peyoruvic acid	71.2	2116896.94 ± 210262.6	3338298.88 ± 126165.75	5769580.95 ± 909796.16	12085739.94 ± 824814.52
2,4-Dichloro-2,5-dihydro-5-oxofuran-2-acetate	64.8	24614024.59 ± 494053.57	18882966.58 ± 2774358.34	10221042.93 ± 2327562.8	10497121.76 ± 112818.11
2-(4-methylthio)butylmalate	231.9	122820.61 ± 18942.84	682559.6 ± 876740.96	4803970.78 ± 2576704.22	11928982.78 ± 2051820.78
2-Hydroxy-6-oxo-6-(2-hydroxyphenyl)-hexa-2,4-dienoate	253.2	862928.72 ± 29253.1	4180531.84 ± 1046122.57	6974197.89 ± 363006.54	10619794.09 ± 1324543.31
2-Hydroxy-3-(4-hydroxyphenyl)propanoic acid	176.8	5194560.48 ± 730992.49	7976667.08 ± 394807.41	14187566.07 ± 1264003.02	22975740.93 ± 10616957.76
2,3,4,5-Tetramethoxybenzoic acid	204.6	188219.31 ± 20992.82	1094170.56 ± 278564.64	1167284.36 ± 98477.46	7961828.66 ± 994781.14
3-[5-acetyl-4-(carboxymethyl)-1H-pyrrol-3-yl]propanoic acid	176.9	6815423.28 ± 259652.43	12517051.16 ± 573102.14	23353550.03 ± 871697.22	21220531.17 ± 309202.13
Methylisocitric acid	75.1	71167939.68 ± 328026.15	56902888.28 ± 1103319.82	47222329.06 ± 4133655	32821078.81 ± 1978539.47
3-(4-Hydroxy-3-methoxyphenyl)-2-methyllactic acid	261.1	481094.53 ± 35299.77	1037143.92 ± 186414.66	1318377.24 ± 81900.86	4738763.79 ± 2459037.26
9-Methylthio-2-nonanoic acid	276.6	504170.07 ± 168545.28	470043.91 ± 13501.72	4002833.16 ± 241962.16	6751667.3 ± 3496862.53
2-Hydroxy-6-oxo-6-(2-hydroxyphenoxy)-hexa-2,4-dienoate	111.1	932344.43 ± 73444.25	1834186.18 ± 83694.54	2394282.21 ± 120627.61	4680926.71 ± 653488.36
N-formylanthranilic acid	236.1	2071140.48 ± 148253.26	2365948.21 ± 9734.92	4093665.79 ± 217650.72	4754124.99 ± 122881.53
4-Fluoro-1,2-dihydroxy-1,2-dihydrobenzoic acid	264.8	225640.38 ± 27149.3	297276.92 ± 21585.01	4018185.43 ± 250648.5	8543853.68 ± 1808687.84
3-Dehydroshikimic acid	214.5	364292.73 ± 180194.35	490842.03 ± 108221.43	4000864.67 ± 865603.35	3896644.69 ± 1392853.51

3-Hydroxyhexadecanoic acid	374.1	548013.09 ± 331560.13	3513566.42 ± 3017675.63	5543663.11 ± 2933522.88	52017389.29 ± 16331686.36
Iminodiacetate (IDA)	176.9	21198082.93 ± 191544.09	33208326.77 ± 1161809.31	58005842.55 ± 1770902.22	49718723.47 ± 1216118.23
Methyloxaloacetate	69.6	24766808.43 ± 1734681.83	7995869.15 ± 763137.45	8378243.6 ± 527711.93	3539973.23 ± 196316.55
4-Hydroxybenzoate	245.9	160914.06 ± 74853.33	231638 ± 195293.93	14167084.15 ± 3757202.56	13567569.17 ± 4895622.69
Hydroxy-methoxy cinnamic acid	83.3	1265007.82 ± 427387.96	18334296.78 ± 10611281.86	18441687.9 ± 4105867.1	40531179.31 ± 13978333.94
8-Dehydro-3-deoxy-D-manno-octulosonate	45.1	1036495.96 ± 783537.01	749383.38 ± 342323.09	18725474.94 ± 4814440.76	26365827.8 ± 8058919.16
2-Hydroxybenzenepropanoic acid	116.7	1234741.03 ± 752242.72	3274568.47 ± 1918248.29	16150865.74 ± 4799844.81	16382862.32 ± 5444623.92
16-oxo-palmitate	351.1	653085.58 ± 174624.97	1942439.93 ± 466938.28	4728146.2 ± 1264712.03	16805700.03 ± 16381269.44
Phenylpyruvic acid	300.6	781177.7 ± 170718.51	1443993.35 ± 36836.96	2128183.57 ± 632776.57	24664585.45 ± 1693561.57
p-Coumaric acid	196.9	1096171.92 ± 66782.88	1722231.09 ± 343834.69	6333291.32 ± 1492225.41	6258909.58 ± 531094.95
**3-[(1E,4R)-4-hydroxycyclohex-2-en-1-ylidene]pyruvic acid**	**225.7**	**2808890.13 ± 75658.54[d]**	**4275199.56 ± 444016.56[c]**	**6754854.21 ± 454504.95[b]**	**11363772.48 ± 340564.34[a]**
Haematommic Acid	79.2	452874.02 ± 216408.06	871770.57 ± 572299.77	13214851.55 ± 3393723.4	37646577.43 ± 11685173.69
4-Oxo-2-azetidinecarboxylic acid	177.1	1295806.19 ± 108784.1	1763318.45 ± 136944	3045649.61 ± 339806.9	2645114.59 ± 94550.29
3-Hydroxytetradecanoic acid	325.8	143300.82 ± 101732.53	1170067.14 ± 839716.72	2509719.23 ± 471420.65	11962478.58 ± 4086053.13
Phenylacetic Acid	93.6	2207067.56 ± 1121971.17	8670301.76 ± 2349281.51	20564968.72 ± 1221654.07	34639367.24 ± 22507828.3
Ibuprofen	249.5	3480297.64 ± 149313.58	7678730.68 ± 242095.36	6183098.92 ± 62225.86	17448922.32 ± 365873.17
**Organic Acids and Derivatives**					
Mycosporine	98	11968983.34 ± 158953.46	15473938.7 ± 859769.58	35407817.86 ± 903468.35	30163640.01 ± 811958.54
6,8-Dihydroxy-7-methoxy-3-methyl-3,4-dihydroisochromen-1-one	143	5681238.68 ± 237757.98	15673431.04 ± 502626.21	27661480.82 ± 681286.63	25196591.03 ± 4242879.44
1-[5-Hydroxy-1-(2-hydroxy-ethyl)-2-methyl-1H-indol-3-yl]-ethanone	318.5	278169 ± 21692.19	566639.85 ± 31559.04	6161182.1 ± 156381.63	10487006.55 ± 4318598.96
Feruloyl allylamine	223.9	4533668.07 ± 22641.48	9153225.03 ± 460661.5	9727332.92 ± 263582.54	11796700.83 ± 644934.46
(3R,4R)-7,2,4,2-tetrahydroxy-4,5-methylenedioxyisoflav-3-ene	49.6	41233833.25 ± 3892008.18	131070171.22 ± 12476869.58	105304288.27 ± 28066439.38	434732778.58 ± 165810340.28
19-Hydroxy-8-O-methyltetrangulol	49.5	2149584.65 ± 920028.44	37633796.8 ± 5319891.17	25549592.97 ± 7115728.29	132981105.78 ± 54880470.45
5-(Hydroxymethyl)-6-(1-hydroxypropyl)-4-methoxypyran-2-one	235.6	195213.55 ± 12667.19	1126831.73 ± 543041.38	1548693.57 ± 56542.9	13063293.63 ± 1317404.64
5'-Deoxyadenosine	270	27664517.2 ± 818643.53	11611663.91 ± 501949.58	10975334.11 ± 2677704.74	7976996.13 ± 1696349.96

Leprotin	425	39904563.63 ± 3501057.24	36022623.78 ± 11078203.34	18370364.3 ± 1719606.4	15332937.44 ± 373842.36
Alternariol monomethyl ether	262.8	468140.85 ± 32589.46	3121831.96 ± 199940.14	3955064.04 ± 131094.32	5895002.13 ± 1076145.27
tolfenpyrad	340.6	176536.85 ± 20003.28	243518.52 ± 52583.27	412574.06 ± 53355.76	422312.06 ± 103906.94
Corynoxidine	65	330493.25 ± 34466.63	360906.97 ± 19157.93	38128.04 ± 6785.47	18157.82 ± 17933.26
Androsin	304.5	96543.01 ± 9767.95	505407.53 ± 74068.46	2544683.65 ± 31166.51	2020091.66 ± 219894.71
Khaephuoside B	425.5	376817412.04 ± 273858248.67	18943683.41 ± 12430820.42	2674118.14 ± 1429721.37	2073730.38 ± 431073.71
O-Methylarmepavine, (R)-	248.6	796512.04 ± 9403.35	4565261.32 ± 397674.94	4278865.13 ± 422370.8	8045185.97 ± 691217.75
Picrotin	333.2	1568331.26 ± 625149.6	4402438.56 ± 2508628.93	5802917.95 ± 1954693.36	20298865.85 ± 7467402.46
Megalomicin A	422.9	29014545.11 ± 2229889.79	19185988.39 ± 14250430.8	1925867.93 ± 1371925.79	782202.23 ± 731156.5
**4-Hydroxyacetophenone**	**251.2**	**362501.91 ± 125484.39[c]**	**5280170.21 ± 364314.9[bc]**	**10119993.17 ± 2810930.97[ab]**	**14510117.66 ± 4935633.9[a]**
**Methylparaben**	**245.2**	**460024.89 ± 103221.57[b]**	**8193143.66 ± 5781048.97[b]**	**17692659.26 ± 5761452.5[b]**	**129040249.91 ± 46433302.71[a]**
SCHEMBL1423113	209.2	5270528.57 ± 200125.69	6595710.94 ± 331900.64	12262635.59 ± 1168123.36	14674432.75 ± 442990.63
(S)-Nandigerine	290.8	89738.8 ± 31481.82	146393.83 ± 1079.9	838708.26 ± 61752.09	7434796.67 ± 2477668.82
Benahorin	230.7	538456.02 ± 12804.08	3085223.86 ± 116223.44	4648895.7 ± 734434.45	16062829.11 ± 4723863.91
Crinamin	208.8	1246440.89 ± 8536.33	4536664.02 ± 102173.3	8007791.87 ± 408207.09	14082280.73 ± 3763942.79
4-Methyl-2-methylene-1-(1methylethylidene)-cyclohexane	321.2	17124292.65 ± 714398.98	7692680.02 ± 417324.34	5763740.28 ± 211137	820044.84 ± 787104.52
Indicine	323.7	1308235.81 ± 61789.84	3356245.82 ± 393310.21	5884166.05 ± 306322.59	6467940.84 ± 500721.23
5'-O-Methylthymidine	155.2	4416717.23 ± 94828.91	4720336.87 ± 378673.24	21805681.6 ± 1028304.02	21337032.98 ± 1424463.28
**Enicoflavine**	**121.8**	**986357.03 ± 27874.69[c]**	**2315685.91 ± 19245.63[c]**	**6549646.58 ± 256827.96[b]**	**19414945.55 ± 4447539.96[a]**
Pectolinaroside	47.2	1918424.71 ± 961887.49	4164367.12 ± 1733711.6	8902718.41 ± 3061519.42	14368382 ± 6797491.24
5-(3-Pyridyl)-2-hydroxytetrahydrofuran	121.1	494841.7 ± 49627.91	699887.73 ± 24152.06	1689532.56 ± 312552.83	12185259.11 ± 3851518.96
Betamipron	268.5	1249401.17 ± 55048.6	5004926.98 ± 1823854.29	6311239.8 ± 110293.06	17586948.73 ± 708409.39
Carbimazole	42.8	25136775 ± 3174033.33	13354343.95 ± 783736.05	7203730.65 ± 817743.77	6357938.29 ± 373021.52
Bamethan	94.3	5827394.27 ± 255860.88	6475807.26 ± 196741.03	54509988.39 ± 3212579.1	55878418.27 ± 1381163.34
**CHEMBL506347**	**296.6**	**3265582.74 ± 131503.69[d]**	**5640971.26 ± 139625.21[c]**	**6884862.58 ± 379711.29[b]**	**10374898.83 ± 449777.92[a]**

Mellein	106.8	3600107.97 ± 1119762.24	4805213.05 ± 120841.72	12571099.42 ± 662255.96	14150002.15 ± 1490992.74
3'-Aminoacetophenone	70.1	33258683.32 ± 725345.65	42508999.21 ± 322600.93	68285077.28 ± 18222007.03	71947038.3 ± 3302250.47
Daphnetol	221.6	720309.94 ± 92003.18	2489003.11 ± 114114.99	7620381.03 ± 45720.05	6182154.2 ± 385839.65
6-Hydroxy-4-methyl-2-benzopyrone	302.4	1485866.9 ± 19631.35	2717289.94 ± 1275229.34	4535428.01 ± 1594001.54	83854940.56 ± 2901848.12
2,4,6-Trimethyl-1,3,5-dioxathiane	51	110601658.73 ± 1311322.93	90772125.13 ± 2251958.83	86549995.13 ± 1937072.23	49022917.45 ± 1074081.32
4-Hydroxy-3,5,6-trimethyl-2H-pyran-2-one	285	577423.46 ± 37765.86	982007.43 ± 35654.88	964950.79 ± 70439.18	2265218.6 ± 128678.11
1-Isothiocyanato-7-(methylthio)heptane	92.6	9056389.68 ± 1011595.21	21775127.96 ± 873965.45	26848843.78 ± 1819725.43	36787820.17 ± 19217372.93
Homoveratrylamine	70.1	10204160.53 ± 263882.26	13672550.99 ± 5163666.73	21134903.75 ± 915386.19	55329813.93 ± 965936.34
Eugenitol	267	624009.06 ± 63055.51	2049447.22 ± 126100.16	1963234.01 ± 127807.9	5828246 ± 94863.09
4-Hydroxychalcone	228.3	195890.66 ± 18736.61	196663.13 ± 15672.56	1063099.79 ± 76936.38	1748949.56 ± 416594.45
1-Nitro-5,6-dihydroxy-dihydronaphthalene	161.6	2028905.58 ± 159965.41	6635944.6 ± 276330.58	17305984.67 ± 1190322.63	17499516.14 ± 586339.47
Piperonal	127.5	6599227.72 ± 137895.47	7427566.74 ± 562051.37	16711715.11 ± 254590.27	17604362.86 ± 826936.88
**Thalifoline**	**254.7**	**802929.37 ± 44324.53[d]**	**2862459.9 ± 75286.85[c]**	**11259683.82 ± 273158.09[b]**	**15592238.66 ± 853099.61[a]**
Betonicine	68.9	13183967.12 ± 743565.81	27183508.36 ± 812571.02	24120891.02 ± 620198.76	35918505.46 ± 1008057.09
CHEMBL1161513	105.5	1144053.74 ± 56558.93	1848913.51 ± 30364.1	8380175.62 ± 3214604.91	19596340.14 ± 9357298.74
3-Methylxanthine	62.1	11418586.17 ± 1987898.04	23399592.27 ± 1771997.17	56940583.87 ± 38518724.46	73458054.64 ± 24962230.81
**Heterocyclic Compounds**					
Homocitrulline	53.9	26247057.47 ± 1040128.07	25650002.32 ± 2129026.79	19572982.01 ± 783278.15	16311928.97 ± 483363.27
delta-Valerobetaine	67	15850959.91 ± 742356.39	53343936.44 ± 1978494.49	56811328.74 ± 2425372.46	131104023.82 ± 3362202.43
**L-Glutamic acid**	**51**	**2036443674.14 ± 7895400.53[a]**	**1644866535.49 ± 21924817.11[b]**	**1584681874.88 ± 23399543.87[c]**	**945011674.23 ± 2902022.41[d]**
Stachydrine	85.1	4820314.85 ± 142967.24	6396448.83 ± 84510.16	9038905.7 ± 631976.38	42988426.43 ± 10630529.49
Threonylglycine	51.6	18886877.7 ± 258784.73	7218526.91 ± 589219.78	8916713.56 ± 163292.35	3580725.94 ± 733878.64
**Cyclo(Tyr-Leu)**	**200.3**	**500146.12 ± 24341.88[d]**	**2033180.41 ± 101476.72[c]**	**5968024.03 ± 900005.03[b]**	**17388462.42 ± 1447666.35[a]**
Beta-Aminobutyric Acid	50.3	122169197.81 ± 2534762.52	200891775.24 ± 10034709.94	177425784.84 ± 5407587.99	364595597.13 ± 9946231.88
N-Acetylleucine	53.6	28381738.52 ± 978560.12	53141276.45 ± 1509180.72	66631068.97 ± 3008724.09	88414116.58 ± 5275672.46

L-2-Amino-3-(4-aminophenyl)propanoic acid	310.2	318549.71 ± 18495.42	565970.06 ± 50433.01	1640551.47 ± 95182.79	2250640.81 ± 128701.84
Fructoseglycine	70.1	15477680.38 ± 628638.7	19492639.44 ± 1568859.12	19407586.94 ± 1094051	27670274.05 ± 1324393.09
Met Ile Val	70.1	10803446.26 ± 795685.68	28266658.27 ± 528986.85	39425216.5 ± 2027712.67	38068809.07 ± 2813628.57
Synephrine	226.2	744126.8 ± 10585.54	776047.49 ± 23414.36	2938988.19 ± 650257.48	23847640.84 ± 1125628.88
2-amino-3-oxo-4-(phosphonooxy)butanoic acid	42.9	3211085.81 ± 1026275.59	3769133.98 ± 846422.08	5071707.72 ± 848024.44	9799206.61 ± 501340.99
Valine	68	183835984.04 ± 4960854.52	319424486.57 ± 5743223.71	322483469.93 ± 15110084.22	1394192587.83 ± 79812481.43
L-Alanyl-L-norleucine	117.2	235309.2 ± 31980.03	1211200.31 ± 619320.58	3753775.39 ± 239143.45	2748128.58 ± 225096.56
5-Aminopentanoic acid	55.1	2265278.92 ± 1384420.19	3312946.15 ± 2947379.3	29219231.58 ± 8707102.95	35185094.06 ± 12112008.35
**L-Theanine**	**125.5**	**591633.61 ± 35526.04[b]**	**530455.28 ± 49461.58[b]**	**2887694.29 ± 629085.37[b]**	**15630189.85 ± 7134274.12[a]**
2-Amino-3-chlorobenzoic acid	75.4	7885607.26 ± 254813.66	4250083.53 ± 218405.67	4374158.32 ± 448529.25	1953810.22 ± 291969.2
N,N-Dimethylarginine	49	176904961.07 ± 2670213.86	177964292.26 ± 7464882.23	124586124.84 ± 2075511.7	97771895.57 ± 2122821.3
O-Methyl-L-Tyrosine	106.9	8022882.49 ± 126847.57	13847620.12 ± 148150.39	38085021.98 ± 1634917.86	42179225.31 ± 576153.86
**Amino Acids and Derivatives**					
1-Methylpyrrolinium	236.6	57300.16 ± 10694.52	2775884.36 ± 49704.91	1893034.12 ± 151860.87	20985513.41 ± 5623123.33
5-Hydroxytryptophol	225.7	741136.12 ± 169176.35	4844674.51 ± 310108.87	6656128.49 ± 607428.34	10822567.14 ± 331974.19
**4,6,8-Trimethyl-quinolin-2-ol**	**266.8**	**216314.52 ± 24963.11[c]**	**379602.49 ± 34333.77[c]**	**1440905.85 ± 118888.72[b]**	**3052875.38 ± 615875.96[a]**
3H-Indole	122	11347875.5 ± 445361.23	16356456.17 ± 240093.3	15076308.69 ± 597672.86	20867002.27 ± 1914901.76
1-methyl-2-phenylquinolin-4(1h)-one	245.3	986430.13 ± 50945.54	3464668.66 ± 330691.69	9101632.04 ± 271885.23	10845667.24 ± 761196.79
Irgarol	221.2	104178.33 ± 12237.99	697352.3 ± 159632.96	1021283.6 ± 108026.9	4063105.11 ± 605912.66
7-Methylxanthosine	104.9	6383437.41 ± 274945.18	26131416.46 ± 1176212.05	27913448.65 ± 603219.64	37430689.05 ± 3925093.47
Nicotinamide riboside	50.5	33165438.78 ± 2075828	27357829.42 ± 1771555.79	23289415.21 ± 2760969.81	10296798.06 ± 135609.42
**Oxyquinoline**	**274.1**	**383802.52 ± 20122.22[b]**	**1305509.95 ± 254108.17[b]**	**6691957.14 ± 1762802.29[b]**	**36352787.2 ± 12423740.15[a]**
N-Methylnicotinamide	219.5	843047.53 ± 60702.58	2499109.12 ± 52826.53	2162002.91 ± 158082.44	8192963.44 ± 824240.55
Simazine	79.8	2927639.33 ± 146144.89	3068877.32 ± 218710.03	20001773.88 ± 1321024.5	3079168469.84 ± 52707624.31
2-propyl-4H-3,1-benzoxazin-4-one	80.7	1271777.05 ± 92091.06	1524127.61 ± 74374.42	2542885.99 ± 49536.56	11418336.86 ± 456611.12
Penciclovir	108.4	12450207.43 ± 334644.49	14085929.91 ± 1430090.1	28639927.06 ± 1400205.05	34231323.99 ± 1130762.12

D-1,2,3,4-Tetrahydronorharman-3-carboxylic acid	215.6	2633460.43 ± 1344862.7	4650496.08 ± 821897.43	23114698.53 ± 63173.05	112171576.54 ± 4022185.05
Phenytoin dihydrodiol	224.9	285618.99 ± 17217.58	857078.8 ± 73821.1	952046.03 ± 132197.79	1281152.48 ± 271776.12
Trenbolone	312.9	347440.02 ± 12524.95	302798.11 ± 14634.58	688842.71 ± 72749.5	1080047.86 ± 107264.81
**Alkaloids**					
1-(9Z-octadecenoyl)-2-heptadecanoyl-glycero-3-phosphocholine	410.3	22911353.86 ± 5086038.32	19048903.53 ± 10713643.43	7278096.11 ± 4955298.22	2312456.34 ± 201321.48
1-Hexadecanoyl-2-(14-pentadecenoyl)-sn-glycero-3-phosphocholine	412	156013342.54 ± 73771659.6	86586213.1 ± 85407721.87	3357006.9 ± 3099230.05	408045.42 ± 279394.74
PC(18_3(6Z,9Z,12Z)_18_4(6Z,9Z,12Z,15Z))	413.8	92711309.63 ± 15375728.53	14332938.32 ± 9737021.66	1020563.37 ± 357544.98	1853283.03 ± 750351.7
PC(22_6(4Z,7Z,10Z,13Z,16Z,19Z)_20_5(5Z,8Z,11Z,14Z,17Z))	440.5	32441997.6 ± 8172990.53	20708005.09 ± 7085030.64	10353401.9 ± 8542328.46	2344482.69 ± 630428.07
1-(1Z-octadecenyl)-2-(9Z-octadecenoyl)-sn-glycero-3-phosphocholine	423.5	133570885.59 ± 171454747.37	26940497.34 ± 4693638.48	3927156.64 ± 501997.3	1074008.5 ± 700865.11
1-Stearoyl-2-arachidonoyl-sn-glycero-3-phosphocholine	440.7	118994554.24 ± 44309639.2	52520126.58 ± 26972346.55	5100771.4 ± 2145671.58	6325317.77 ± 1665050.55
1-Stearoyl-2-linoleoyl-sn-glycero-3-phosphatidylcholine	396.5	68317262.79 ± 26984890.69	47604886.31 ± 6568666.39	24613189.54 ± 7963195.05	16173783.04 ± 5544628.09
Glycerophosphoglycerol	47.6	2787660.57 ± 622800.67	4371606.45 ± 616572	16362874.38 ± 5488586.32	14811126.46 ± 3064775.51
1-Palmitoyl-lysophosphatidylcholine	389.8	61766331.65 ± 15708399.94	131684134.47 ± 72871696.36	231781674.32 ± 6777675.43	764375191.35 ± 33166781.43
PC(22_6(4Z,7Z,10Z,13Z,16Z,19Z)_22_6(4Z,7Z,10Z,13Z,16Z,19Z))	424.5	90973552.83 ± 85314541.56	11553973.58 ± 7122493.73	2253159.99 ± 1082788.85	697597.22 ± 215538.8
C2-ceramide	355.3	1044377.52 ± 51391.33	1068006.69 ± 351146.71	7714369.38 ± 232055.82	8356880.86 ± 494109.4
1-Myristoyl-sn-glycero-3-phosphocholine	354.2	83264.22 ± 12146.35	789172.09 ± 59940.86	793451.19 ± 386324.43	2882807.84 ± 394928.91
PC(18_1(11Z)_22_5(4Z,7Z,10Z,13Z,16Z))	418.4	292147511.78 ± 136095430.84	7364608.5 ± 2360938.93	3616565.25 ± 3493198.63	886957.23 ± 344329.08
**Lipids**					
N-Acetylmuramate	70.1	2398875.21 ± 752968.2	3262406.17 ± 142346.2	6184996.97 ± 335765.15	9473593.98 ± 827632.65
Gastrodin	95.3	5654535 ± 42497.42	6417601.96 ± 3221752.44	30368920.99 ± 2532027.89	36166566.2 ± 332912.87
Udp-glucosamine	56.6	25116327.21 ± 3847783.5	17318141.05 ± 2815064.07	14419132.61 ± 2631688.12	8144751.5 ± 2508722.52
3-Ketosucrose	296.7	19942083.56 ± 4211286.74	2964968.06 ± 394965.08	945443.67 ± 571357.56	170556.24 ± 124373.88
N-Acetyl-D-Mannosamine	52.7	37609634.17 ± 613138.98	44798961.03 ± 2060162.42	53935219.35 ± 2656299.69	70444969.35 ± 3101866.38
6-Acetyl-D-glucose	51.1	41946370.07 ± 2202355.46	98468160.54 ± 2116576.95	101584859.83 ± 33107319.46	232584180.69 ± 69312896.22

**Carbohydrates and Derivatives**					
5,9_6,9-Diepoxy-3-megastigmene	291	4103255.11 ± 144522.26	10585997.72 ± 2801700.28	12957069.22 ± 412373.43	15524223.44 ± 2103921.34
5,9_6,9-Diepoxy-4-megastigmene	254.4	464846.2 ± 34070.65	8224276.85 ± 154594.3	10235460.81 ± 2542566.71	17247355.17 ± 7123770.27

Vomifoliol	225	240063.41 ± 19933.83	1310681.34 ± 593859.61	2026696.58 ± 1150462.53	7072372.58 ± 2599384.64
Achillin	286.8	620064.6 ± 52689.24	1231206.54 ± 45756.93	1261422.1 ± 103347.64	5251240.18 ± 442737.35
sinulaflexiolide A	396	2309032.09 ± 1527949.87	20637187.37 ± 19378592.36	52234177.8 ± 14045958.03	64357432.19 ± 34804623.51
Celastrol	392.4	680204.57 ± 73023.53	3578091.39 ± 806923.52	6227453.19 ± 3351942.58	8191222.84 ± 1006238.31
Ecdysterone 2,3_20,22-diacetonide	446.5	74090772.37 ± 21021760.66	57305540.92 ± 16241596.27	20493827.58 ± 2011841.84	19713815.59 ± 4186522.65
Pokeberrygenin	449.6	83764119.42 ± 25666024.33	59594561.03 ± 512875.14	18243793.58 ± 7816622.92	18479579.69 ± 1744230.78
Panaxcerol C	411.7	154522613.8 ± 59430575.65	71305797.19 ± 19685412.67	4471604.62 ± 1669310.74	4095050.93 ± 1785881.79
3-Oxo-5alpha-steroid	289.7	3022391.24 ± 139925.07	2002216.53 ± 62601.01	1349210.79 ± 62300.92	1566230.83 ± 97453.94
miltiradiene	326.4	22239955.47 ± 504856.44	21111104.71 ± 2806656.73	7529720.97 ± 768574.92	7154658.3 ± 1682946.69
**Terpenoids**					
2,3,4,6-Tetrahydroxybenzophenone	75.3	5284441.64 ± 387444.14	3966392.61 ± 171880.46	2993217.74 ± 175990.56	1894179.53 ± 72903
1,3-Benzenediol	119.9	5311011.92 ± 169932.46	17338071.1 ± 8820642.58	19242426.13 ± 776859.51	65475945.68 ± 30805985.26
Chavicol O-beta-glucopyranoside	248.8	607248.74 ± 12571.55	1955982.4 ± 1566420.95	3017130.83 ± 155163.25	90907291 ± 16880715.53
5-Phenyl-1,3-oxazinane-2,4-dione	253	421489.31 ± 96651.09	549948.2 ± 327458.99	1492388.06 ± 73431	6508212.22 ± 2885913.71
2,5-Dimethylresorcinol	273.2	298348.49 ± 24042.42	1959017.19 ± 1097532.54	2237913.1 ± 366128.44	7018649.66 ± 5995204.32
(3-Ethoxyphenyl)-(2,3,4-trihydroxyphenyl)methanone	269	1114286.51 ± 337918.35	4276773.3 ± 165864.29	3874136.11 ± 785741.98	12624746.16 ± 315733.1
3,5-Dimethoxyphenol	86.5	2054099.04 ± 1907687.3	8205584.3 ± 4421114.9	10624521.1 ± 928801.09	32872668.52 ± 1344495.8
1-Phenyl-2-pentanol	256.5	627475.31 ± 10830.03	3007844.72 ± 121073.95	3669092.36 ± 190830.36	4935892.25 ± 236502.04
Tiliroside	285.8	6380128.07 ± 2914044.35	4113937.01 ± 325336.03	1149411.41 ± 409111.29	715535.76 ± 616908.35
Hyperforin	388.6	662801.74 ± 23917.5	6914441.73 ± 4074588.48	5461895.34 ± 168037.28	22955553.78 ± 6537178.98
Apigenin 7-[rhamnosyl-(1->2)-galacturonide]	444.7	18149955.63 ± 4270318.18	1789969.74 ± 465843.8	547255.33 ± 27325.57	569834.88 ± 321274.53
6''-O-(3-Hydroxy-3-methylglutaroyl)astragalin	418.3	440408593.4 ± 210846517.34	9027334.4 ± 265737.67	4827659.21 ± 4537455.9	2506720.73 ± 693358.48
3,5,2-Trihydroxy-7,8-dimethoxyflavanone	209.5	1155841.06 ± 121625.38	1066796.83 ± 35178	861750.89 ± 66104.97	460812.09 ± 24101.45

Okanin 4-methyl ether 4-O-(6-O-p-coumaroylglucoside)	430.5	381932683.03 ± 114568234.4	39305955.02 ± 22768982.67	4791733.94 ± 3247034.9	2416149.02 ± 401894.49
Griseoxanthone C	250.7	812921.18 ± 15225.55	677081.37 ± 102650.03	3202430.95 ± 122640.17	6362597.85 ± 786977.04
3-Methoxy-2-(4-methylbenzoyl)-4H-1-benzopyran-4-one	283.8	1517470.27 ± 103228.12	1692261.59 ± 131747.49	1982209.92 ± 178509.04	3673029.64 ± 98076.64
6-Formylumbelliferone	118.3	3167368.46 ± 338252.58	1124065.38 ± 303464.6	1021649.12 ± 89929.37	170543.34 ± 30491.82
Bergapten	49.8	56719830.66 ± 4454731.64	84694555.28 ± 1543246	118339828.69 ± 29542936.88	477798076.06 ± 156026723.61
4-Hydroxycoumarins	244.6	715502.23 ± 120769.43	715863.07 ± 40020.77	2380174.86 ± 344542.92	11091087.65 ± 1286633.87
**Phenols**					
4-Oxo-1-(3-pyridyl)-1-butanone	67	1532003.66 ± 807882.45	4631345.72 ± 236962.46	5675145.96 ± 284332.85	29163364.08 ± 972945.75
**Ketones**					
Phytosphingosine acetamide	355.6	425908.6 ± 78574.16	467101.65 ± 254065.66	3481887.76 ± 213483.63	38007704.24 ± 5041448.54
Trimethobenzamide	249.5	1465625.29 ± 40701.77	2225603.34 ± 136317.55	2426355.8 ± 164846.69	2801144.7 ± 586328.79
Isophosphamide mustard	72.7	142275190 ± 6794823.07	77315854.35 ± 1210918.14	55046775.95 ± 6731938.56	50656116.29 ± 5089169.38
**Amides**					
6-Ethyl-2,4-dihydroxy-3-methylbenzaldehyde	226	2234521.02 ± 78574.16	12027235.24 ± 238746.64	12246887.8 ± 1179757.45	12360156.62 ± 92108.38
2,5-Furandicarbaldehyde	55.7	6009113.31 ± 40701.77	13433413.41 ± 841584.83	13506264.92 ± 1622112.89	12517724.44 ± 314848.08
Syringaldehyde	258.2	1527618.27 ± 6794823.07	83235808.29 ± 44393867.17	86871359.09 ± 644214.86	83967249.15 ± 1365502.16
N-Formyl-Met-Leu-Phe	284	1336630.01 ± 96651.09	82248.93 ± 5317631.02	39462.12 ± 6007402.91	48820.69 ± 269300.11
**Aldehydes**					

Results are presented as mean ± standard deviation. Columns with different letters indicate a significant difference ($p < 0.05$) according to Duncan's multiple range test. The different superscript letters (a-e) indicate a significant difference at the 0.05 significance level ($p < 0.05$). RT: retention time.

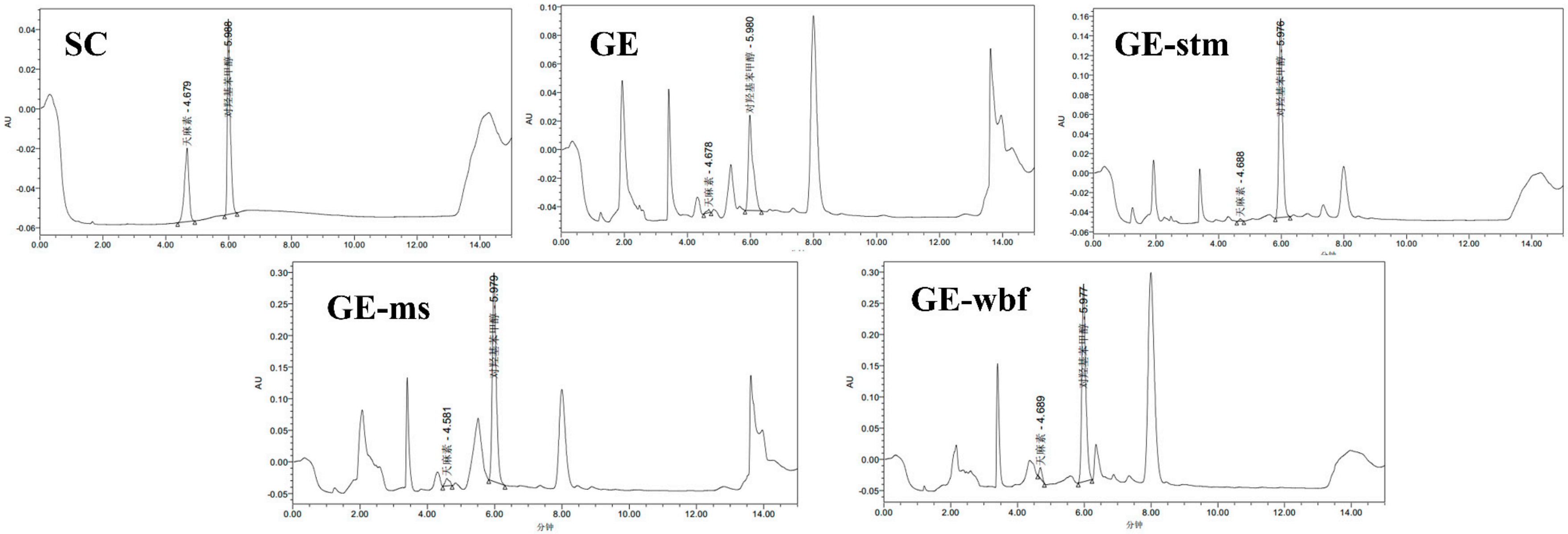

Figure S1. Representative HPLC chromatograms of gastrodin and p-hydroxybenzyl alcohol in fresh and processed Gastrodia elata. (A) GE, (B) GE-stm, (C) GE-ms, and (D) GE-wbf. Peaks 1 and 2 correspond to gastrodin and p-hydroxybenzyl alcohol, respectively. GE, fresh *Gastrodia elata*; GE-stm, steamed *Gastrodia elata*; GE-ms, moistened *Gastrodia elata*; GE-wbf, bran-fried *Gastrodia elata*.
